# Giving Ideas Some Legs or Legs Some Ideas? Children’s Motor Creativity Is Enhanced by Physical Activity Enrichment: Direct and Mediated Paths

**DOI:** 10.3389/fpsyg.2022.806065

**Published:** 2022-03-10

**Authors:** Nicoletta Tocci, Patrizia Scibinetti, Emiliano Mazzoli, Myrto Foteini Mavilidi, Ilaria Masci, Mirko Schmidt, Caterina Pesce

**Affiliations:** ^1^General Psychology Laboratory, Department of Movement, Human and Health Sciences, University of Rome “Foro Italico”, Rome, Italy; ^2^Institute for Physical Activity and Nutrition, Faculty of Health, School of Exercise and Nutrition Sciences, Deakin University, Geelong, VIC, Australia; ^3^Early Start, School of Education, University of Wollongong, Keiraville, NSW, Australia; ^4^Institute of Sport Science, University of Bern, Bern, Switzerland; ^5^Exercise and Cognition Laboratory, Department of Movement, Human and Health Sciences, University of Rome “Foro Italico”, Rome, Italy

**Keywords:** divergent movement, creative thinking, executive function, development, physical education, constraints-led approach, cognitive stimulation

## Abstract

Approaches to foster motor creativity differ according to whether creative movements are assumed to be enacted creative ideas, or solutions to emerging motor problems that arise from task and environmental constraints. The twofold aim of the current study was to investigate whether (1) an enriched physical education (PE) intervention delivered with a joint constraints-led and cognitive stimulation approach fosters motor creativity, and the responsiveness to the intervention is moderated by baseline motor and cognitive skills and sex; (2) the intervention may benefit motor creativity through gains in motor coordination, executive function, and creative thinking. Ninety-five children, aged 6–9 years, participated in a 6-month group randomized trial with specialist-led enriched PE vs. generalist-led conventional PE. Before and after the intervention, Bertsch’s Test of Motor Creativity, Movement Assessment Battery for Children, Random Number Generation task and Torrance Test of Creative Thinking were administered. Linear mixed models were run accounting for the random effects of data clusters. Multiple mediation analysis was performed to assess whether motor coordination, executive function and creative thinking mediated any improvement of motor creativity. Results showed that (1) specialist-led enriched PE, compared to generalist-led conventional practice, elicited a more pronounced improvement in all motor creativity dimensions (fluency, flexibility, and originality) independently of baseline levels of motor and cognitive skills and sex; and (2) improved motor creativity was partially mediated by improved motor coordination and, as regards motor flexibility, also by improved inhibitory ability. In conclusion, enriching PE with tailored manipulations of constraints and variability may enhance the ability to create multiple and original task-pertinent movements both directly and through indirect paths. The results are discussed extending to motor creativity a theoretical framework that distinguishes different creativity modes. The intervention may have fostered the generation of creative movements directly through the exposure to variation in constraints, activating the sensorimotor ‘flow’ mode of creativity that bypasses higher-order cognition, but also indirectly through a systematic and conscious convergence on solutions, activating the ‘deliberate’ mode of creativity that relies on inhibition to reject common or task-inappropriate movement categories.

## Introduction

Physical activity (PA) is increasingly acknowledged by exercise scientists as an investment in human capital, with PA outcomes framed as capitals in the physical, mental, and socio-emotional domains (Bailey et al., 2013). Similarly, creativity researchers point to the age of human capital we are living in and propose that creativity is the currency of the modern era (Kell et al., 2013). At the crossroad of these two perspectives on the role of physical activity and creativity to build human capital lies the domain of proficient and creative movement.

In the last decade, there has been a flourishing of research suggesting the centrality of motor competence for children to develop the full potential of their multifaceted capital (van der Fels et al., 2015; Barnett et al., 2021; Pesce et al., 2021a). However, within the ongoing debate on what is motor competence (Bardid and Utesch, 2018) and how it should be assessed (Eddy et al., 2020) and trained (Jiménez-Díaz et al., 2019), motor competence is mostly conceived as proficient performance of fundamental movement skills, such as running or throwing, and tested by observing the reproduction of predefined movement skill patterns or measuring their efficacy. Recent attempts to broaden the scope of motor competence have proposed to shift the focus from ‘fundamental’ to ‘foundational’ skills. They encompass also ‘non-fundamental’ movement skills considered important for promoting PA (e.g., cycling; Hulteen et al., 2018), or a wider range of variable skills that emerge through exploration and detection of opportunities for action (Ng and Button, 2018) also referred to as ‘functional’ movement solutions (Rudd et al., 2021a).

This shift from discrete fundamental movement skills performed under stable conditions to multiple nested movement skills performed in complex and fluid situations fits within the broader framework of physical literacy. Beyond the usual definition as the competence, confidence and knowledge to be physically active for life, a recent overarching model highlights the holistic nature of physical literacy and the reciprocal and reinforcing relations of motor competence with knowledge, motivational, affective and social processes that emerge from the person-context interaction in various real life settings (Cairney et al., 2019). Parallel to and partly driven by this extension of the meaning of motor competence, motor creativity – the ability to produce functional or expressive solutions to movement tasks that are novel, original, and pertinent (Memmert, 2011) – and related constructs, such as movement variability (Orth et al., 2017; Pesce et al., 2019), adaptability (Richard et al., 2018b), functional novelty (Hristovski et al., 2011) and exploration (Stodden et al., 2021) – are gaining momentum. Nevertheless, interventional evidence on how to foster it is limited.

Theory-based interventional research performed in sport and gross-motor performance arts (such as dance) suggests that motor creativity is sensitive to tailor-made intervention programs in both adult skilled performers (e.g., Memmert, 2011; Torrents et al., 2015) and children and adolescents (e.g., Rasmussen and Østergaard, 2016; Santos et al., 2017; Thomaidou et al., 2021). In early childhood education, movement-based creativity programs have been employed to foster not so much motor creativity *per se*, but creative thinking in the embodied and kinaesthetic way that characterizes discovery learning and cognitive development in the early years (Grammatikopoulos et al., 2012). In school-aged children, evidence of benefits of motor creativity programs in physical education (PE), though promising, mostly lacked a theoretical base (Bournelli, 1998; Bournelli and Mountakis, 2008; Chatoupis, 2013) with few exceptions (Richard et al., 2018a).

Richard et al. (2018a) compared the effects two PE programs (conventional vs. creative) on multiple dimensions of motor creativity and creative thinking. The creative motor program included the same content of the conventional program but was delivered according to non-linear pedagogical principles in PE drawn from the Ecological Dynamics theory (Rudd et al., 2021b). Non-linear pedagogy focuses on the interaction between the learner and the environment that constrains their action and is characterized by teaching strategies that exploit functional movement variability. This differs from teaching with linear pedagogy, which relies on repeated, model-oriented skill practice (Rudd et al., 2021b). Richard et al. (2018a) found that the creative motor program improved fluency and flexibility in moving, and originality in thinking. To interpret the effect on originality in thinking but not in moving, the authors suggested that children’s motor experience and skills might have been insufficient to translate increased original ideas into original movements, or their cognitive control insufficient to suppress the tendency toward performing more common movements.

Evidence on the relation between motor creativity and motor coordination development in children is mixed, showing either a positive association (Milić, 2014; Santos and Monteiro, 2021), or no association (Scibinetti et al., 2011; Marinšek and Lukman, 2021). Also the role of cognition in children’s motor creativity is an open issue. On the one side, creative movements are hypothesized to be enacted creative ideas. This hypothesis is consistent with cross-sectional associations between creative thinking and creative sport performance (Santos et al., 2017; Santos and Monteiro, 2021) and underlies the suggestion that designed PA programs may foster motor creativity in children through the development of divergent cognitive processes (Bournelli and Mountakis, 2008). On the other side, creative solutions to motor problems are suggested to arise from task and environmental constraints without any abstract rule representation of the emerging creative behavior (Hristovski et al., 2011; Orth et al., 2017). This view is supported by evidence on working memory – a cognitive executive function enabling to hold and update task-relevant information in mind, which seems unrelated to the generation of creative movement actions by children (Scibinetti et al., 2011) and adults (Furley and Memmert, 2015; Moraru et al., 2016) but involved in the divergent generation of creative thoughts (De Dreu et al., 2012). Instead, inhibition – an executive function allowing to control interference and suppress routine thoughts and behaviors – seems related to the ability to produce original movements (Scibinetti et al., 2011), whereas it is unclear whether inhibition (Khalil et al., 2020) or disinhibition (Radel et al., 2015) is involved in creative thinking.

The primary aim of this study was to verify whether a theory-based enriched PE program, grounded on the constraints-led approach and variability of practice (Tocci and Scibinetti, 2007; Richard et al., 2018a; Pesce et al., 2019; Scibinetti, 2019) may aid children’s motor creativity. We also verified whether children’s responsiveness to the motor creativity intervention may be enhanced in presence of a high baseline level of creative thinking, as hypothesized by Richard et al. (2018a), or higher baseline levels of motor coordination and executive function, and be different in males and females. Secondarily, we explored whether improved motor coordination, executive function (particularly inhibition) and creative thinking mediate improvements in motor creativity dimensions. We hypothesized two potential paths (Dietrich, 2019). On the one hand, the manipulation of constraints might lead to creative perception-action couplings, relying on motor coordination and bypassing higher-order cognition and consciousness. On the other hand, enhanced inhibition might support a deliberate mode of creativity (Dietrich, 2019) and convergent creative processes (Zhang et al., 2020), allowing to converge on creative solutions by refraining from more common ones. Inhibition is a multifaceted construct as early as childhood (Huizinga et al., 2006). We targeted a specific facet of the inhibition construct – cognitive inhibition (Diamond and Ling, 2020), which is the ability to inhibit routine thoughts and memories and seems related to motor creativity (Scibinetti et al., 2011). We hypothesized that if creative motor behaviors are enacted ideas, then cognitive inhibition may help suppress thinking routines that would not allow diverging from habitual ideas and behaviors.

## Materials and Methods

The study is part of a broader longitudinal research program approved by the Ethics Committee of the “Umberto I” hospital of the First Rome University (Ref. No 2950) and authorized by the school Committees and students’ parents, who gave written informed consent. We did not seek child assent, as this study was part of regular PE classes.

### Study Design

In a class-randomized trial, eight classes were randomly assigned to either an intervention of specialist-led enriched PE designed to foster motor creativity or generalist-led conventional PE. The participants were tested during the curricular school time on primary motor creativity outcomes (fluency, flexibility, originality in moving, and overall motor creativity) and secondary outcomes potentially contributing to motor creativity in the motor domain (motor coordination [evaluated as motor impairment scores]: manual dexterity, aiming and catching skills, balance, and overall motor impairment) and in the cognitive domain (creative thinking: fluency, flexibility, originality in thinking, and overall creative thinking; executive functions: inhibition and working memory) at baseline and after 6-month intervention, corresponding to the end of the school year. At baseline, demographic information on age, sex (assigned at birth), body weight and height, outdoor play and structured physical activity/sport training was also collected.

### Participants

Participants were 142 primary school children, recruited from eight (1st to 4th grade) classes of two urban schools in the municipality of Alba (in the Northern of Italy). Within each school, one class for each school grade was stratified randomly included in the study and the two participating schools were randomly assigned to the intervention or control condition. The regional and local PE coordinators invited school principals to participate within the broader frame of a whole-child initiative of PA promotion supported by a Public-Private-Partnership. School principals and teachers were provided information on the aim of the study and offered in-person presentation, and principals were invited to provide written organizational consent. Following school recruitment, a school liaison person from each school (identified by the consenting school) was provided with a plain language descriptions of the study and a consent form for parents/guardians.

**FIGURE 1:**
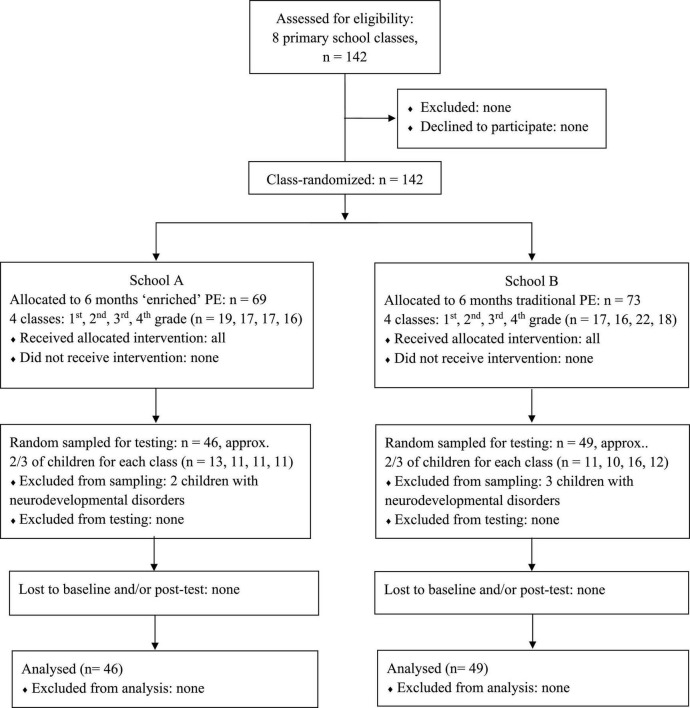
Study flow diagram for the class-randomized controlled trial.

To collect data within the time constraints for test administration posed by the participating schools and at the same time limit the cluster effect of the class-based recruitment, two-thirds of the 142 children were selected from each of the eight classes with systematic random sampling, with cluster size ranging from 10 to 16 children (*M* = 12 ± 2). Thus, the final sample comprised 95 children aged 6–9 years (*M* = 7.8 ± 1.3). The progress through the phases of enrolment, intervention allocation, and final sample for data analysis is represented in Figure 1. Children with certified neurodevelopmental and/or mental health conditions (e.g., children diagnosed with mild intellectual-relational disability or developmental learning disorder) were excluded from within-class random sampling to avoid too large deviations in the dataset. This applied to two in the intervention classes and three in the control classes. Further demographic characteristics such as socio-economic status, considered sensitive information by the schools, could not be assessed.

### Intervention

#### Content and Delivery

The intervention was designed in a theory-based manner, including key elements of two theoretical approaches grounded on different assumptions, yet complementary for our purposes: the constraints-led and the cognitive stimulation approach. For this integration, we relied on emerging evidence on the advantage of hybridizing pedagogical models that seems best suited to promote outcomes in multiple domains, overcoming the boundaries of single theoretical approaches (González-Víllora et al., 2018).

According to the principles of the constraint-led approach, the primary role of the teachers involved in the intervention was not that of an instructor, who aims at modeling children’s movement skills, but rather that of a facilitator of the interaction of the learners with the environment through the purposeful manipulation of environmental and task constraints. In line with Orth et al.’s (2017) viewpoint, we designed a progressive manipulation of the environmental and task constraints to facilitate children’s exploration of both new coordination solutions (i.e., different movement patterns, corresponding to the flexibility dimension of creativity) and new control solutions (i.e., different parametrizations of the same movement pattern, contributing to the fluency dimension of creativity). As an example of environmental constraints, obstacles of different size/height may generate different affordances (i.e., opportunities for action in the environment) for children to produce different solutions to overcome the obstacles, either exploring new coordination solutions (e.g., vaulting instead of jumping if the child may rely on adequate skills and the obstacle surface allows vaulting supported by the hands), or exploring a new control solution (e.g., changing some joint angles to jump over a higher obstacle without substantially changing the jump coordination). As regards task constraints, we adopted both direct and indirect releasing (Hristovski et al., 2011). Directly releasing constraints means using less stringent instructions that increase the affordances that can satisfy constraints; indirectly releasing means using more stringent instructions that, while hindering more common solution pathways, direct the learners in otherwise unexplored directions and promote the perception and utilization of new affordances. As an example of indirect releasing of task constraints, constraining the parts of the body that can touch the floor while moving (e.g., supporting the body on only one hand and one foot, or no feet, or neither hands nor feet) leads children to explore new locomotor coordination solutions beyond the common bipodalic ones.

Thus, our methodology embraced the viewpoint that a constraints-led exploration may enhance the variability of functional movement patterns and promote divergent movement ability directly rather than through the enactment of an antecedent creative idea (Orth et al., 2017), in line with a flow mode of creativity that relies on sensorimotor coupling rather than higher-level cognition (Dietrich, 2019). However, it has been suggested that a creative act may not be the manifestation of only one creativity mode, as we can also evaluate, select and converge on creative solutions by means of a deliberate mode of creativity that relies on top-down cognitive control (Dietrich, 2019). Thus, we complemented the constraints-led approach with the cognitive stimulation approach. To operationalize in the motor domain the stimulation of cognitive control processes, we adopted principles of variability of practice applied in motor learning with linear and non-linear pedagogies, as both provide opportunities to generate cognitive engagement (Pesce et al., 2019). Linear pedagogy relies on the classical theory of motor learning stages that conceives learning as a progression from an initial cognitive stage to a final autonomous stage of maximal movement automaticity and minimal cognitive engagement (Schmidt et al., 2011). Following Tomporowski et al. (2010), teachers of the intervention classes introduced systematic changes to the motor learning tasks to generate contextual interference and new cognitive challenges, and keep children ‘on the learning curve.’ To foster the deliberate, cognitively engaging mode of creativity, teachers also manipulated the time constraints on the search for solutions and asked children to select the rarest and most original ones within different time frames. This was assumed to challenge cognitive inhibition, which is the inhibition of routine thoughts and memories (Diamond and Ling, 2020) and children’s awareness that we tend to produce habitual movement actions if there is not enough time to inhibit most common ideation solutions and evaluate the originality of different emerging alternatives. These principles were applied in designed PA games. Two sample games with game alterations and task analysis to zoom into the game demands are presented in Supplementary Data Sheet 1. The first game (“Magnets and mechanisms”) is an example of constraint-led approach and non-linear pedagogy to foster the search for solutions to satisfy constraints with a focus on cooperation and cooperative creativity, which are meaningful goals of school education (Torrents et al., 2021a). The second game (“A friend is a treasure”) is an example of hybridization of pedagogical models. It is provided in two versions: the first is targeted to cognitive stimulation through systematic variations of coordinative demands applied with both linear and non-linear pedagogy; the second version adds further manipulation of task and time constraints along with teaching through questions to foster awareness of the creative process.

Instead, generalist teachers of the control classes were instructed to perform their ‘business as usual’. Within the Italian primary education, PE lessons are traditionally conducted by generalist and not specialist teachers. Generalists, though having competence in student-centered pedagogy, during their formal tertiary training do not receive specific training on how to use a student-centered pedagogy concretely for delivering PE lessons. Their scarce knowledge, competence and confidence in the own PE teaching skills translates into ‘conventional’ PE lessons characterized by mainly prescriptive tasks and teaching strategies primarily driven by teachers’ safety concerns and control efforts, rather than mastery-oriented strategies that require exploration and a certain degree of risk-taking.

#### Setting, Duration, Blinding, and Fidelity

The intervention was performed in the gym or sports court of the school during the curricular PE time for 1 h once a week, as prescribed by school regulation, and lasted 6 months from November to April with a total amount of 24 intervention hours divided into four 6-week teaching modules. The teacher–child ratio was about 1:18 in the control classes but was altered in the intervention classes, where a specialist PE teacher delivered the intervention in the presence of the generalist classroom teacher; however, this latter did not actively participate to the PE delivery except for a limited supportive role for individual children. Due to the presence of the PE specialist, teacher and children could not be blinded with respect to the assignment to generalist-led or specialist-led PE but were blinded as to the expected outcomes.

To ensure implementation fidelity but also an adequate degree of adaptability, PE specialists used a handbook, which describes the pedagogical principles and the PA games designed to foster motor creativity, with each game including several alterations to help teachers adapt the games to their children’s skills and needs (Pesce et al., 2016a). The teaching materials also included sheets with tree diagrams to identify the task demands of each game in different domains and how they were altered in the game variations. Moreover, PE specialists underwent a 6-h teacher training every 6 weeks and participated to regular group discussions with the generalist teachers of their intervention classes. These group discussions were aimed to align the contents of each teaching module across classes and discuss teaching issues arisen in the previous module, as well as to foster generalists’ learning of the enriched PE methodology for future application after the end of the intervention. During training, PE specialists were taught how to use the handbook and the tree diagram sheets for task analysis, and how to create nuanced game variations according to the pedagogical principles outlined in the handbook. No adverse events or side effects occurred in specialist-led enriched or generalist-led conventional PE classes.

### Assessment Instruments and Procedures

Measurement tools were selected according to following criteria: (1) evidence of validity and reliability; (2) space and time requirements appropriate to ensure feasibility in the ecological PE context. All tests were administered in the school setting during the curricular school time. Detailed information on assessment tasks, validity and reliability are reported with reference to primary articles in Supplementary Data Sheet 2.

#### Primary Outcome: Motor Creativity

Children’s motor creativity was assessed with the Bertsch’s test (1983). This test is composed of four tasks to be performed individually, in randomized order on the floor, with a bench, a hoop or a ball, respectively. These tasks are available in two separate versions (form A and B) varying in the degrees of freedom of the movement tasks, with form A providing no specific performance modality and form B partially defining it. For this study, we used the Bertsch’s test form B. During each task, children’s motor behavior was video recorded. The tasks lasted 2 min and 30 s (hoop and floor) or 3 min (ball and bench) for a total test duration of about 20 min including the initial instruction and breaks between tasks (for more information: Bertsch, 1983; Scibinetti et al., 2011).

##### Motor Creativity Tasks

###### Floor

Two parallel lines delimited a 2.5-m^2^ area on the floor. The verbal instruction was: “Your task is to move from one line to the other. You are free to do anything you want between these two lines. Show me all possible ways you know or that may come to your mind to do that.”

###### Bench

A bench was located in the middle of a room, with two hoops positioned at the two ends of the bench representing the starting and arrival point. The verbal instruction was: “You have to go from one hoop to the other and back, keeping a part of your body always in contact with the bench.”

###### Hoop

Two parallel lines representing the starting and arrival point were put at 3.5 m. The verbal instruction was: “Your task is to move the hoop from one line to the other. You can let it go on its own or take it with you. Show me anything you can do that comes to mind.”

###### Ball

Children were situated in the middle of a 2.5-m^2^ square. Their task was to use a ball to hit seven 1-m^2^ targets outside the square and positioned on the wall, floor, and ceiling, one at a time. The verbal instruction was: “You see all the targets around you. Imagine they are glasses. Try to break them with this ball without going out of your home (i.e., the square). What’s important is not so much to break all the glasses but to try to break them every time in a different way.”

##### Data Coding and Scoring

Motor behaviors were coded and scored by a blinded expert investigator. A further blinded investigator independently coded a subsample of motor behaviors and inconsistencies were solved by consultation (inter-observer agreement rate > 80%). Data coding consisted in assigning scores on three dimensions: fluency, flexibility, and originality. Fluency was scored in terms of the number of different behaviors displayed by the child during each motor task. Flexibility and originality were coded and scored based on Bertsch’s (1983) normative data. Flexibility was scored referring to movement behavior categories identified by Bertsch for each task (16 for the hoop and the ball tasks, 44 for the bench task, and 36 for the floor task). Such categories summarize motor behaviors as a function of body position, movement direction and type. The flexibility score was the sum of movement categories, identified as a function of body position, movement direction and type, for which at least one behavior was observed, categories with two or more observed behaviors being counted only once. Originality was quantified assigning a score ranging from 0 (low originality) to 3 (high originality) to each behavior in each category based on the relative frequency of such behavior in Bertsch’s normative sample. To obtain a total score for each creativity dimension, scores for the four tasks were standardized and averaged. Furthermore, a grand average of creative thinking at pre and post-test was computed by merging the three creative thinking variables.

#### Secondary Outcomes in the Motor Domain: Motor Coordination

Children’s motor coordination was assessed individually with the Italian version of the Movement Assessment Battery for Children (M-ABC; Henderson and Sudgen, 1992). The more recent version (M-ABC-2, Schulz et al., 2011) was not available to the researchers. This test is composed of three subheadings: manual dexterity (three tasks), aiming and catching skills (two tasks), and static and dynamic balance (one and two tasks, respectively), with the eight tasks differentiated in four age-related difficulty levels (for more information: Henderson and Sudgen, 1992; Croce et al., 2001).

##### Motor Coordination Tasks

###### Manual Dexterity

Based on children’s age, the first task was ‘posting coins,’ ‘placing pegs,’ or ‘shifting pegs by rows’: the second task was ‘threading beads,’ ‘threading lace,’ or ‘threading nuts on bolt’; and the third task was ‘bicycle trail’ or ‘flower trail.’

###### Aiming and Catching Skills

Based on children’s age, the first task was ‘catching bean bag,’ ‘one-hand bounce and catch,’ or ‘two-hand catch’; and the second task was ‘rolling ball into goal’ or ‘throwing bean bag into box.’

###### Static and Dynamic Balance

Based on children’s age, the task evaluating static balance was ‘one-leg balance,’ ‘stork balance,’ or ‘one-board balance’; the first task evaluating dynamic balance was ‘jumping over cord,’ ‘jumping in squares,’ or ‘hopping in squares’; and the second one was ‘walking heels raised,’ ‘heel-to-toe walking,’ or ‘ball balance.’

##### Data Coding and Scoring

For each of the three subheadings, data were transformed into impairment scores of motor function according to age-related normative data (Henderson and Sudgen, 1992). Then, the three scores were summed up to obtain a total impairment score, indicating the extent to which a child falls below the level of his/her age peers.

#### Secondary Outcomes in the Cognitive Domain: Executive Function and Creative Thinking

##### Executive Function

###### Random Number Generation Task

In the Random Number Generation (RNG) task, version validated for children aged 5 years and older (Towse and McIachlan, 1999), children were tested individually and instructed to verbally generate a random sequence of numbers between 1 and 10 to each beat of a 70-beat sequence with an inter-beat interval of 1.5 seconds. They were presented the RNG as a game involving numbers with a game-like instruction. Both the omission of a number generation in correspondence to one tone and the production of numbers < 1 or >10 were considered errors and discarded. The 70-number generation sequence was preceded by an identical familiarization trial. The whole test lasted about 6–8 min (for more information: Towse and Neil, 1998; Towse and McIachlan, 1999).

###### Inhibition and Working Memory Indices Computation

The randomness of the generated numbers was estimated by means of 18 different indices (Towse and Neil, 1998). Six of them were selected: three reflecting the ability to inhibit mental counting routines [turning point index (TPI), adjacency score (Adj), runs score (Runs)], and three the ability to update information held in working memory [redundancy score (Red), coupon score (Coupon), and mean repetition gap (MeanRG)]. All indices were standardized (i.e., *z*-scores) and average indices of inhibition and working memory were computed. Since high levels of TPI and MeanRG, but low values of Adj, Runs, Red, and Coupon reflect a good inhibition and working memory updating ability, Adj, Runs, Red, and Coupon were reversed before averaging (for more information see: Miyake et al., 2000; Audiffren et al., 2009).

##### Creative Thinking

The Italian version of the Torrance Test of Creative Thinking (TTCT), Figural Form A (Torrance, 1988), designed for individuals in kindergarten through graduate school and beyond, was group administered. It consists of three timed pencil and paper picture construction and completion activities lasting 10 min each with 1 min break between tasks for a total working time of about 30 min (for more information: Torrance and Ball, 1984; Torrance, 1988; Cramond et al., 2005; Kim, 2006).

###### Creative Thinking Tasks

###### Activity I: Picture Construction

Children had to construct a picture using a darkened curve shape (jellybean or teardrop) provided on a page as a stimulus to be integrated in the picture construction.

###### Activity II: Picture Completion

Children had to use 10 incomplete figures to make a figure or object drawings to the incomplete figures, avoiding usual and obvious completions.

###### Activitiy III: Parallel Lines

Children had to use 30 pairs of straight lines drawn on three pages to make an original picture out of each pair of lines.

###### Data Coding and Scoring

Torrance Test of Creative Thinking pictures were coded and scored by a blinded expert investigator based on three sub-scales of norm-referenced measures: fluency, flexibility, and originality. A further blinded investigator independently coded and scored a subsample of pictures (inter-observer agreement rate > 80%). Fluency was scored by the number of figural images produced by the child; flexibility by the variety of categories of relevant responses; and originality by the number of statistically infrequent responses based on normative data (Torrance, 1988). Raw scores were converted into standard scores to have comparable ranges for fluidity, flexibility, and originality. Furthermore, a grand average of creative thinking at pre and post-test was computed by merging the three creative thinking variables.

#### Demographic Variables

At baseline, children’s body mass and height were measured for body mass index (BMI, kg/m^2^) computation. Children’s spontaneous play habits in outdoor environments were estimated by means of the Children’s Outdoor Play assessment questionnaire (Veitch et al., 2009; Italian validation: Pesce et al., 2016b). Parents reported the number of days their child spent at least 10 min playing in locations such as their yard at home, a friend’s or neighbor’s yard, their street or court or footpath, a park or playground in out-of-school hours on weekdays (eight items on a five-point scale) and weekend days (eight items on a six-point scale) during a typical week. Parents also answered few questions regarding their children’s actual practice (e.g., number of days/week, session duration) of after-school sports or any other structured PA training (for more information: Veitch et al., 2009; Pesce et al., 2016b).

### Preliminary Analyses

#### Manipulation Checks

Manipulation checks were used to ensure that PE ‘enrichment’ in the intervention classes was operationalized by teachers with teaching strategies that truly involved problem solving, guided and divergent discovery, and cognitive challenges, and to evaluate to what extent these qualitative delivery characteristics were independent from or coupled with different levels of enjoyment.

##### Teaching Strategies

All intervention and control classes were video recorded during a representative PE session for analysis of teaching behaviors. The lesson was recorded at about midpoint of the intervention period. To ensure representativeness, no indication was given except that the lesson should not deviate from usual PE praxis (e.g., it should not be devoted to the preparation of a special sport-related or cultural event). The qualitative features were categorized by two independent experienced raters as behavioral categories of teaching strategies and quantified by means of event sampling as percentage (%) of events for time unit (20 s). A satisfactory inter-observer reliability (>80%) was reached. The behavioral categories of teaching strategies used for the analysis were (Rink, 2006): (1) Interactive teaching (instructional process controlled by teacher); (2) peer teaching (reciprocal feedback and evaluation by students); (3) cognitive strategies (teaching through questions, problem solving, guided, and divergent discovery); (4) cooperative learning (achievement of meaningful goals through teamwork).

Specialist PE teachers in the intervention classes exerted control over the entire instructional process less frequently than generalist teachers in the control classes (interactive teaching: 23% vs. 87% of events) and used teaching strategies in a more differentiated way, shifting from themselves to the children specific decisions/responsibilities along the instructional process (Mosston and Ashworth, 2008). Expectedly, specialists frequently used cognitive strategies (47%), mainly based on problem solving with convergent and divergent discovery (45%), only rarely used by generalists (3%). Specialists also used peer and cooperative teaching (20 and 11%, respectively) more frequently than generalists (10 and 0%, respectively).

##### Physical Activity Enjoyment

The PA Enjoyment Questionnaire (Di Cagno et al., 2006) comprised six semantic differential items (e.g., anchored by “pleased/unpleased,” and “enjoyed/bored”) with a 5-point picture-based Likert scale evaluating whether the child enjoyed the PA tasks composing the PE lesson. The specialist-led enriched PE group showed a slightly higher average enjoyment score than the generalist-led conventional PE group (*M* = 4.67 ± 0.41 vs. 4.28 ± 0.45), as emerged from the non-parametric Mann–Whitney test applied to the negatively skewed data (*U* = 580, *n* = 95, *p* < 0.001) (for more information: Di Cagno et al., 2006).

#### Design Effect

Since children in the intervention and control groups were clustered in eight classes, with observations within each cluster being not independent, the cluster design effect was computed and used as a multiplier of sample size determined with *a priori* power analysis. The design effect computation takes into account that the variance of the mean computed from a clustered sample is larger by a factor of [1 + (*n* − 1) * ICC], modified to consider differences in cluster size (i.e., number of children tested in each class) as follows:

$$\text{Cluster}\,\text{effect}=\left\{1+\left[\left({\text{CV}}^{2}+1\right)\times \text{n}-1\right]\times \text{ICC}\right\}$$

where *n* = number of children within each cluster, CV = coefficient of variation for n and ICC = intraclass correlation coefficient [σ^2^ between-cluster/(σ^2^ between-cluster + σ^2^ within-cluster)]; Hedges and Hedberg, 2007). Given the absence of ICC reference values for the primary outcome of motor creativity and the low ICC reference values available for the secondary outcomes (motor coordination and executive function: 0.04 and 0.02, respectively; Aadland et al., 2019), we used a conservative estimate of ICC recommended in previous research on PA effects on children’s motor and cognitive development (ICC = 0.15, Resaland et al., 2015). With a mean cluster *n* = 11.87 (±1.88), a CV = 0.16 and the conservatively assumed ICC = 0.15, the estimated cluster design effect was 2.68. This design effect value was used as a multiplier of sample size determined with *a priori* power analysis for α = 0.05, β-1 = 0.80 and the minimal detectable effect size (ES [*f*, i.e., $\eta_{\text{p}}^{2}$**/(1 −** $\eta_{\text{p}}^{2}$)]) = 0.26 for motor fluency and 0.23 for motor flexibility according to Richard et al.’s (2018a) findings. The estimated sample size to detect intervention effects on motor fluency and flexibility was 86 and 102, respectively. Our sample size (*n* = 95) was between these estimates.

## Results

### Preliminary Analyses

Table 1 presents children’s demographics and background characteristics, as well as pre- and post-intervention values of primary and secondary outcome variables separately for group and sex. Mahalanobis distance was computed to identify multivariate outliers in the outcome variables used for analysis. Two outliers were identified, with *p* of Mahalanobis distance < 0.001. Main analyses were run both with and without outliers. Since the pattern of results remained substantially unchanged, the outliers were maintained.

**TABLE 1:** Demographics, spontaneous outdoor play and structured sports training, and pre- and post-intervention values of primary outcome variables (motor creativity: fluency, flexibility, and originality in moving) and secondary outcome variables in the motor domain (motor coordination [evaluated as motor impairment scores]: manual dexterity, aiming and catching skills and balance) and in the cognitive domain (executive function: inhibition and working memory; creative thinking: fluency, flexibility, and originality in thinking) of 6–9 year-old children assigned to the specialist-led enriched or generalist-led conventional physical education (PE).

Group			Specialist-led enriched PE		Generalist-led conventional PE	
***N***			46		49	
**Sex** (*n* males/*n* females)			23/23		24/25	
**Age** (years)	Pre-intervention		7.7 ± 1.2		7.8 ± 1.4	
**Body mass index (BMI)** §	Pre-intervention		17.8 ± 2.9		18.0 ± 3.0	
Lean [*n* (%)]			30 (65%)		35 (71%)	
Overweight [*n* (%)]			16 (35%)		14 (29%)	
**Spontaneous outdoor play** (score ± *SD*)	Pre-intervention		33.6 ± 9.6		34.4 ± 10.8	
**Structured sports training** (min/week ± *SD*)	Pre-intervention		126 ± 95		126 ± 92	
**Motor creativity** (std score ± *SD*)			Males	Females	Males	Females
Fluency		Pre	0.05 ± 0.81	−0.31 ± 0.67	−0.18 ± 0.84	−0.32 ± 0.69
		Post	0.51 ± 0.72	0.42 ± 0.54	−0.02 ± 0.72	−0.10 ± 0.73
Flexibility		Pre	0.01 ± 0.74	−0.28 ± 0.61	−0.18 ± 0.82	−0.39 ± 0.65
		Post	0.52 ± 0.75	0.42 ± 0.47	0.06 ± 0.74	−0.11 ± 0.64
Originality		Pre	0.19 ± 0.74	−0.24 ± 0.60	−0.16 ± 0.84	−0.31 ± 0.54
		Post	0.45 ± 0.77	0.35 ± 0.64	0.01 ± 0.72	0.24 ± 0.53
**Motor coordination** (impairment score ± *SD*)			Males	Females	Males	Females
Manual dexterity		Pre	5.26 ± 3.43	5.07 ± 3.32	7.79 ± 3.70	5.81 ± 2.97
		Post	3.72 ± 3.15	3.48 ± 3.02	4.95 ± 3.38	4.65 ± 3.51
Aiming/catching skills		Pre	2.89 ± 2.66	3.61 ± 2.96	2.59 ± 2.67	3.23 ± 2.96
		Post	0.57 ± 1.30	1.41 ± 2.25	1.70 ± 2.16	1.62 ± 2.39
Static/dynamic balance		Pre	4.78 ± 3.56	2.26 ± 3.13	3.74 ± 2.94	3.06 ± 3.25
		Post	1.41 ± 1.76	1.91 ± 3.56	1.99 ± 2.06	1.77 ± 2.05
**Executive Function** (std score ± *SD*)			Males	Females	Males	Females
Inhibition		Pre	−0.51 ± 0.99	−0.23 ± 1.11	−0.38 ± 1.04	0.07 ± 1.04
		Post	0.42 ± 0.48	0.35 ± 0.69	0.26 ± 0.49	0.01 ± 0.78
Working memory		Pre	0.22 ± 0.50	0.03 ± 1.08	−0.16 ± 0.93	−0.35 ± 0.84
		Post	−0.02 ± 0.70	0.09 ± 0.60	0.04 ± 0.49	0.18 ± 0.64
**Creative thinking** (score ± *SD*)			Males	Females	Males	Females
Fluency		Pre	16.52 ± 8.69	19.16 ± 6.17	16.99 ± 5.82	20.31 ± 6.39
		Post	21.13 ± 7.12	25.03 ± 7.66	22.32 ± 7.38	24.28 ± 6.13
Flexibility		Pre	14.09 ± 7.01	14.57 ± 4.41	12.98 ± 4.67	16.49 ± 5.04
		Post	17.74 ± 5.02	18.75 ± 5.36	15.60 ± 4.97	18.34 ± 4.06
Originality		Pre	16.65 ± 9.85	16.62 ± 8.15	16.07 ± 7.80	20.61 ± 6.42
		Post	24.13 ± 9.94	26.42 ± 9.66	21.05 ± 9.39	22.92 ± 8.24

*^§^ Lean vs. overweight status based on age-referenced cut-off values of BMI (Cole et al., 2000).*

#### Group Differences at Baseline

One-way ANOVAs with group as factor were performed on demographic and PA variables (age, spontaneous outdoor play, structured physical activity/sport training), primary outcome variables (fluency, flexibility, and originality in moving) and secondary outcome variables in the motor domain (motor coordination [evaluated as motor impairment scores]: manual dexterity, aiming and catching skills, static and dynamic balance) and in the cognitive domain (executive function: inhibition and working memory; creative thinking: fluency, flexibility, originality in thinking). Group differences were found only in working memory [*F*(1,93) = 4.22, *p* = 0.043, $\eta_{\text{p}}^{2}$ = 0.04] and manual dexterity [*F*(1,93) = 85.52, *p* = 0.021, $\eta_{\text{p}}^{2}$ = 0.06], with the intervention group showing a higher baseline working memory performance and manual dexterity (i.e., lower impairment score) as compared to the control group.

#### Correlations of Outcome Variables at Baseline

Spearman’s correlation coefficients computed to estimate the level of association, at baseline, of motor creativity with the other outcome variables (motor coordination, executive function, and creative thinking) are reported in Table 2 for the entire sample and separately for males and females. Results showed significant weak to moderate correlations of all three dimensions of motor creativity (fluency, flexibility, originality) with a majority of dimensions of motor coordination and creative thinking, and with inhibition but not working memory. Correlations were overall stronger in males than females, only males showing significant correlations of motor creativity with creative thinking and inhibition. Sex differences in correlation were statistically tested: they were significant only for manual dexterity with motor fluency (*z* = 1.73, *p* = 0.042), and inhibition with motor fluency (*z* = −1.78, *p* = 0.037) and flexibility (*z* = −1.91, *p* = 0.028).

**TABLE 2:** Correlations (Spearman’s Rho) at baseline of motor creativity with the other outcome variables in the motor (motor coordination) and cognitive domain (executive function and creative thinking).

Baseline	Motor coordination (impairment score)			Executive function (std score)		Creative thinking (score)		
			
Motor creativity (std score)	Manual dexterity	Aiming/catching	Static/dynamic Balance	Inhibition	Working memory	Fluency	Flexibility	Originality
Fluency	#			#				
Females	−0.01	−0.33*	−0.17	0.07	−0.12	0.09	0.21	0.15
Males	−0.36*	−0.47*	−0.36*	0.42*	−0.02	0.26	0.39*	0.41*
All	−0.16	−0.40*	−0.20*	0.22*	−0.04	0.14	0.27*	0.27*
Flexibility				#				
Females	−0.06	−0.35*	−0.20	0.13	−0.10	0.02	0.14	0.15
Males	−0.34*	−0.56*	−0.41	0.49*	−0.03	0.29	0.42*	0.45*
All	−0.18	−0.45*	−0.23*	0.28*	−0.02	0.13	0.27*	0.30*
Originality								
Females	−0.12	−0.37*	−0.22	0.17	−0.15	0.05	0.13	0.10
Males	−0.36*	−0.52*	−0.41*	0.40*	0.01	0.22	0.39*	0.41*
All	−0.21*	−0.43*	−0.24*	0.26*	−0.03	0.10	0.24*	0.25*

*Significant (p < 0.05); ^#^significant difference in correlation between females and males.

### Main Analyses

#### Analyses and Results of Intervention Effects

To test the hypothesis of intervention effects, we used linear mixed models (LMM). Fixed effects were computed for group (specialist-led enriched vs. generalist-led conventional PE), time (pre vs. post) and their interactions. Separate LMM were run on fluency, flexibility and originality in moving and in thinking, on inhibition and working memory, and on manual dexterity, aiming/catching skills and balance. Random effects were computed to account for clustering of children in classes. Age and baseline values of PA session enjoyment, which resulted higher in the intervention group (see Section “Group differences at baseline”) were included as covariates.

To test the hypothesis that a higher baseline level of motor coordination, executive function, or creative thinking might influence the intervention effects on motor creativity, these four variables were individually included as moderators in separate runs in four further LMM that were run on motor creativity variables (2 Groups × 2 Times × 2 Baseline Motor Coordination or Inhibition or Working Memory or Creative Thinking). To this aim, the grand averages of creative thinking and executive function were dichotomized to obtain binary low vs. high level variables, and the motor impairment scores were used to create, according to M-ABC normative data, a binary variable of typical vs. atypical (borderline movement problems or developmental coordination disorder, DCD) motor development. Moreover, considering some evidence of a higher motor originality of males with the ball (Tocci et al., 2004) that fits with the consistent evidence of males’ superiority in object-control skills (Barnett et al., 2016), a fourth LMM was run adding the factor sex as a moderator.

Planned pairwise comparisons (*t*-tests) were run in the case of significant interactions and effect sizes (Cohen’s *d*) were computed for significant pairwise differences. Bonferroni correction was applied to account for three comparisons (*p* < 0.016) in the *post hoc* analysis of two-way Group × Time interactions (pre-post comparisons separately for the specialist-led enriched and generalist-led conventional PE group and between-groups comparison at post-test) and six comparisons (*p* < 0.008) for three-way interactions with each additional moderator.

##### Primary Motor Creativity Outcomes

**FIGURE 2:**
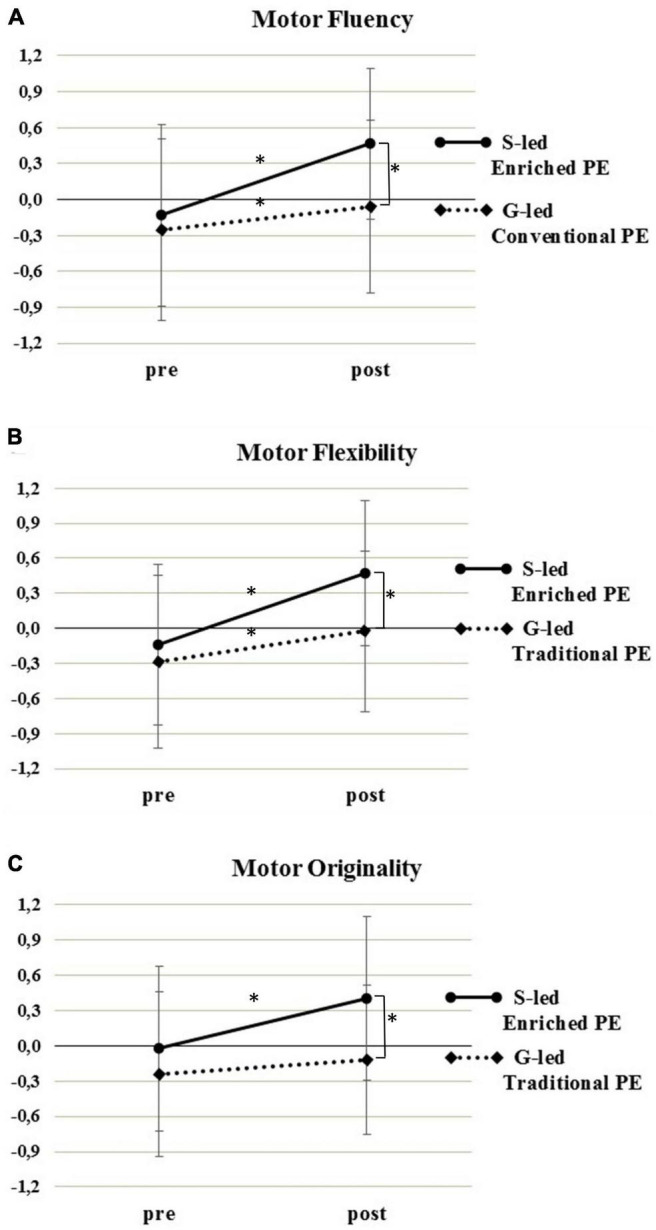
Motor creativity [fluency – panel **(A)**; flexibility – **(B)**; originality – **(C)**] assessed with Bertsch’s test of motor creativity before (pre) and after (post) a specialist-led (S-led) enriched PE intervention or generalist-led (G-led) conventional PE. *p < 0.016 (adjusted for three comparisons).

For all three dimensions of motor creativity, there were a main effect for Time [fluency: *F*(1,94) = 44.36, *p* < 0.001; flexibility: *F*(1,95) = 55.62, *p* < 0.001; originality: *F*(1,95) = 25.41, *p* < 0.001] and a significant Group × Time interaction (Table 3). *Post hoc* comparisons showed a significant pre-to-post increment of motor fluency and flexibility in both groups, more pronounced in the specialist-led enriched PE group than in the generalist-led conventional PE group, leading to a significant group difference at post-test (Figures 2A,B). For originality, the pre-to-post improvement was significant in the enriched PE group only, leading to a significant group difference at post-test (Figure 2C).

**TABLE 3:** Results of main and *post hoc* analyses: group [specialist-led enriched PE intervention group, IG vs. generalist-led conventional control group, CG] × Time [pre vs. post] interactions.

Group × Time	*F* (df), *p*	ICC	*t* (df), *p*, *Cohen’s d*		
			IG pre vs. post	CG pre vs. post	IG vs. CG at post
**Motor creativity**					
Fluency	10.59 (1,95), 0.002	0.15	−6.42 (45), <0.001, 0.69	−2.54 (48), 0.015, 0.03	3.78 (93), <0.001, 0.70
Flexibility	7.44 (1,95), 0.008	0.13	−7.42 (45), <0.001, 0.74	−3.20 (48), 0.002, 0.04	3.66 (93), <0.001, 0.72
Originality	7.70 (1,95), 0.007	0.19	−5.22 (45), <0.001, 0.58	n.s., 0.099	3.80 (93), <0.001, 0.62
**Motor coordination**					
Manual dexterity	n.s., 0.380	0.12			
Balance	n.s., 0.434	0.05			
Aiming and catching	3.23 (1,95), 0.044	0.21	5.71 (45), <0.001, 0.95	4.00 (48), <0.001, 0.49	n.s., 0.116
**Executive function**					
Inhibition	5.94 (1,95), 0.017	0.04	−4.44 (45), <0.001, 0.50	n.s., 0.080	n.s., 0.056
Working memory	5.75 (1,95), 0.018	0.02	n.s., 0.571	−2.51 (48), 0.016, 0.49	n.s., 0.546
**Creative thinking**					
Fluency	n.s., 0.354	0.13			
Flexibility	n.s., 0.061	0.17			
Originality	6.60 (1,95), 0.012	0.19	−5.42 (45), <0.001, 0.13	−3.12 (48), 0.003, 0.04	n.s., 0.117

Adding to the model each of the dichotomous baseline motor and cognitive variables in separate runs, main effects of these variables emerged without the hypothesized significant three-way interaction with Group and Time (*p-*values ≥ .245). The effect of typical/atypical motor development at baseline was significant for motor fluency [*F*(1,92) = 15.68, *p* < 0.001], flexibility [*F*(1,93) = 12.58, *p* = 0.001], and originality [*F*(1,93) = 12.37, *p* = 0.001]; typical motor development was associated with higher motor fluency (difference in std. score between children with typical/atypical motor development [Δ *z*-score] = 0.52), higher motor flexibility and originality (Δ *z* scores: 0.44 and 0.43, respectively). The effect of inhibition at baseline was significant for motor flexibility [*F*(1,91) = 6.32, *p* = 0.014] and originality [*F*(1,93) = 5.52, *p* = 0.021], but not for fluency (*p* = 0.143); higher baseline inhibition was associated with higher motor flexibility (Δ *z*-score between children with low/high inhibition = 0.28) and higher motor originality (Δ *z*-score = 0.28). Working memory at baseline did not affect motor fluency, flexibility, and originality (*p-*values ≥ 0.443). The effect of creative thinking at baseline was significant for motor fluency [*F*(1,83) = 8.53, *p* = 0.005], flexibility [*F*(1,94) = 7.95, *p* = 0.006], and originality [*F*(1,94) = 8.70, *p* = 0.004]; higher baseline creative thinking was associated with higher motor fluency (Δ *z*-score between children with low/high creative thinking = 0.36), higher motor flexibility and originality (Δ *z*-scores = 0.32 and 0.34, respectively). The effect of sex at baseline only approached significance for motor originality (*p* = 0.066, with males tending to be generally more original than females) but did not differentially influence the size of the intervention effect in males and females. There was no significant three-way Group × Time × Sex interaction for any of the motor creativity dimensions (*p*-values ≥ 0.147).

##### Secondary Motor Coordination Outcomes

For manual dexterity, there were neither a main effect for Time (*p* = 0.851), nor a significant Group × Time interaction. For static and dynamic balance, there was only a main effect for Time [*F*(1,95) = 7.40, *p* = 0.008], but no significant Group × Time interaction. For aiming/catching skills, instead, there were both a main effect for Time [*F*(1,95) = 10.64, *p* = 0.002] and a significant Group × Time interaction (Table 3). *Post hoc* comparisons showed a significant pre-to-post amelioration of aiming/catching skills in both groups (Table 3), which was more pronounced in the specialist-led enriched PE group than in the generalist-led conventional PE group (Δ impairment score = −2.26 vs. −1.24); however, the two groups did not significantly differ at post-test (Table 3).

##### Secondary Executive Function Outcomes

For inhibition, there were both a main effect for Time [*F*(1,95) = 20.40, *p* < 0.001] and a significant Group × Time interaction (Table 3). *Post hoc* comparisons showed that the pre-to-post improvement was significant only in the specialist-led enriched PE group (Table 3; Δ *z*-score = 0.75). For working memory, there was only a significant Group × Time interaction (Table 3). *Post hoc* comparisons showed an improvement in the conventional PE group (which approached significance [*p* = 0.016] after applying the Bonferroni correction [adjusted *p* < 0.016]) and no group difference at post-test because the traditional PE group, being worse at baseline, merely caught up over time (i.e., regression to the mean).

##### Secondary Creative Thinking Outcomes

For fluency and flexibility in thinking, there was a main effect for Time [fluency: *F*(1,95) = 50.00, *p* < 0.001; flexibility: *F*(1,95) = 34.41, *p* < 0.001] but no significant Group × Time interaction (Table 3). For originality in thinking, instead, there were both a main effect for Time [*F*(1,95) = 39.52, *p* < 0.001] and a significant Group × Time interaction (Table 3). *Post hoc* comparisons showed a significant pre-to-post amelioration of originality in thinking in both groups, more pronounced in the specialist-led enriched PE group than in the generalist-led conventional PE group (Δ originality score = 8.64 vs. 3.61); however, the two groups did not significantly differ at post-test (Table 3).

#### Analyses and Results of Mediating Mechanisms

In the case of enriched PE effects on both motor creativity and other motor and cognitive skills that might mediate them, multiple mediation analyses were performed with PROCESS macro for SPSS (Hayes, 2013). Specifically, regression analyses were performed on pre-, post-intervention, and pre-post delta data to assess the effects of: (1) the independent variable (X: PE intervention type) on the dependent variable (Y: individual motor creativity dimensions); (2) the independent variable on each mediator (M: total motor impairment, inhibition, working memory, total creative thinking); (3) the independent variable (X) and the potential mediators (M) on the dependent variable (Y). The potential mediators were entered simultaneously in the regression equation to include the covariances among them and the independent variable and verify whether their introduction (i.e., total indirect effect of X on Y through Ms) reduced the direct effect of the PE intervention on the motor creativity dimensions. Bootstrapping was applied to empirically estimate the sampling distribution of the indirect effect and generate 95% confidence intervals (CI).

**FIGURE 3:**
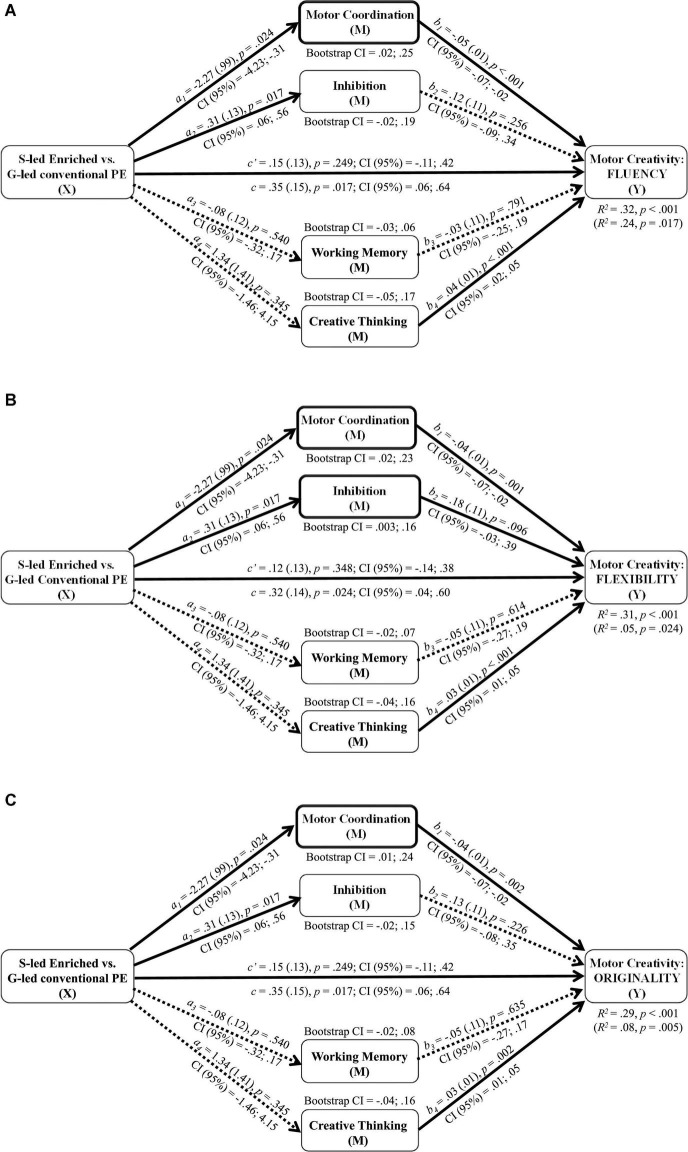
Multiple mediation model: effects of PE group (‘X’: specialist-led [S-led] enriched vs. generalist-led [G-led] conventional) on post-intervention motor creativity [Y: fluency – panel **(A)**; flexibility – **(B)**; originality – **(C)**] mediated by post-intervention level of motor coordination (all motor creativity dimensions) and of inhibitory ability (flexibility only). *a, b, c*: regression coefficients with (SE), *p* and CI (95%) values. *c*: total effect; *a*_1_*b_1_, *a*_2_*b_2_, *a*_3_*b_3_, *a*_4_*b_4_: indirect effects; *c*′: direct effect after accounting for mediators. *R*^2^ values with/(without) mediators and bootstrap CI (95%) for indirect effects are also reported. Solid lines: significant paths; dotted lines: non-significant paths. *Note*. Panel ‘b’: the path linking Inhibition to Flexibility reached significance (*b*_2_ = .22(.11), *p* = .037; CI (95%) = .01; .44) after removing the two non-significant mediators from the model. CI of bootstrap estimates of the indirect effect of Group on Flexibility through Inhibition: .01; .19).

Results showed that the difference in post-intervention improvement of motor fluency, flexibility, and originality between children who participated in the specialist-led enriched or generalist-led conventional PE was partially mediated by the extent to which children ameliorated their total motor coordination (i.e., diminished their total motor impairment score; Figures 3A–C). Only the group difference in post-intervention improvement of motor flexibility was also partially mediated by the extent to which children ameliorated their inhibitory ability (Figure 3B). The path linking Inhibition to Flexibility (panel ‘b’) reached significance after removal of the two non-significant mediators (working memory and creative thinking) from the model (see note of Figure 3). Significant mediation results are indicated by the 95% CI of bootstrap estimates of the indirect effect, which did not include the zero value. The same mediation models applied to pre-intervention and to pre-post delta values did not yield any significant mediation result.

## Discussion

The primary aim of this study was to verify the efficacy of an enriched PE intervention that integrates two different yet complementary theoretical approaches – the constraints-led (Tocci and Scibinetti, 2007; Orth et al., 2017; Scibinetti, 2019; Torrents et al., 2021b) and the cognitive stimulation approach (Tomporowski et al., 2015b; Pesce et al., 2019). The specialist-led enriched PE program, as compared to generalist-led conventional PE, led to more pronounced improvements and higher post-intervention values of motor creativity in all its dimensions, but benefited creative thinking limitedly to its originality dimension. The improvement in motor creativity was not influenced (moderated) by the baseline level of its potential motor and cognitive prerequisites (motor coordination, executive function, and creative thinking) or by sex, but was partially explained (mediated) by improved motor coordination, suggesting that the enriched PE fostered the ability to use improved motor skills to explore the solution space. Moreover, the improved flexibility in moving was jointly explained by motor coordination and cognitive inhibition, suggesting that this latter may prevent from routine thoughts and actions and allow to exploit environmental affordances and motor skills for expanding the solution space to different movement categories.

Evidence on motor creativity promotion is at the core of the motor competence discourse in its broadest meaning and of those interventional strategies that involve a tailored exploration of movement opportunities in the person-context interaction (Stodden et al., 2021). An optimal frame for this discourse is that of physical literacy. Indeed, the notion of engaging ‘positive challenges’ in the exploration of opportunities for action is common to both physical literacy and motor creativity (Jefferies, 2020). Creative problem solving and decision making exercised in PA and sport when coping with movement challenges under varying constraints is proposed to contribute to the development of motor creativity and physical literacy (Rudd et al., 2020), which in turn may lead to positive physical, mental and social health outcomes (Cairney et al., 2019).

### Enriched Physical Education Enhances Creativity in Moving and Originality in Thinking

The primary finding of improved ability to discover many different motor solutions (fluency, Figure 2A) and to make a differentiated and flexible use of various movement categories (flexibility, Figure 2B) to deal with open-ended tasks corroborates previous findings of studies that employed a constraint-led approach and divergent discovery teaching (Chatoupis, 2013; Richard et al., 2018a). Nevertheless, Richard et al.’s (2018a) data presented baseline differences and a pattern of pre-to-post change that could include in the expected intervention effect also a regression to mean (for fluency) or a ceiling effect (for flexibility). Our results reinforce more univocally the interpretation of the differential pattern of pre-to-post change between groups in terms of intervention effect, as we found similar motor creativity gains as Richard et al. (2018a) but without differences at pre-test and with significant differences in favor of the specialist-led enriched PE group at post-test.

Thus, the manipulation of constraints seems a viable way to release degrees of freedom and capitalize on movement variability to produce multiple and diversified movement solution. According to Hristovski et al. (2011), we alternated less and more stringent constraints that hinder common routes and orient the discovery of new affordances. Counterintuitively, constraints may foster the emergence of multiple, diversified and original solutions that are pertinent to solve a motor problem at hand (Torrents et al., 2021b). This apparent paradox of ‘constraining to release degrees of freedom’ in motor behavior is discussed from an Ecological Dynamics perspective and insightfully depicted with the quotation of the Russian composer Igor Stravinsky (The poetics of music) we wish to echo (Torrents et al., 2021b, p. 340): “*My freedom will be so much the greater and more meaningful the more narrowly I limit my field of action and the more I surround myself with obstacles. Whatever diminishes constraint diminishes strength. The more constraints one imposes, the more one frees one’s self of the chains that shackle the spirit.*”

In our study, manipulation checks provided a supportive ‘quantification’ of the fidelity of the qualitative characteristics of the intervention in terms of teaching strategies (Rink, 2006). Children in the specialist-led enriched PE classes experienced problem solving with convergent and divergent discovery much more often than children in generalist-led conventional PE classes. Discovery learning strategies are inherent in the constraints-led approach, though not overlapping with it, as they are rooted in different pedagogical theories. Both motor creativity programs focused on the manipulation of constraints (Richard et al., 2018a) or on divergent discovery teaching strategies (Chatoupis, 2013) were able to foster fluency and flexibility in moving similar to the benefits obtained in the present study.

Our findings also confirmed those by Richard et al. (2018a) in relation to creative thinking, showing an intervention benefit only for the dimension of originality in thinking. Since the children in the intervention group of Richard et al.’s (2018a) study exhibited a high baseline level of fluency and flexibility in thinking, the authors speculatively hypothesized that this might have enabled them to exploit the stimuli provided by the enriched PE intervention to improve motor creativity. We tested this hypothesis and did not find support for a moderating role of baseline creative thinking level, nor of other potential cognitive and motor prerequisites of motor creativity. We found that being less or more creative in thinking, less or more able to inhibit routine thoughts, being with typical or atypical motor development are features associated with children’s motor creativity, but do not impact their ability to respond to motor creativity training. Consistent with previous developmental and adult research on motor and sports creativity (Scibinetti et al., 2011; Furley and Memmert, 2015; Moraru et al., 2016), we found working memory being unrelated to motor creativity.

The gain in original thinking in the present study was paralleled by improved originality in moving (Figure 2C). This might be attributable to the fact that we adopted principles of variability of practice, applied with both linear and non-linear pedagogies that have been proposed to stimulate both thinking and motor skills (Pesce et al., 2019), thus contributing to the increased ability to think originally and perform fluent, flexible and original movements. The beneficial effect on originality may also be due to the manipulation of the time constraints on the search for solutions. From an Ecological Dynamics perspective, constraints act at different time scales, with the selected affordance being the temporarily most attractive one (Torrents et al., 2021b). Extending the time frame available to produce new motor solutions under given task constraints may have enhanced the probability that other less immediate affordances could be selected and more original sensorimotor solutions be produced with a flow mode of creativity (Dietrich, 2019). From a cognitive perspective, extending the time frame of the creative search may have allowed for comparatively slow strategic planning (Tomporowski et al., 2015a). This, rather than rapid online processing, is likely needed for preparing to solve motor problems, evaluate the originality of emerging alternatives and then monitor the progress toward a specific goal with a deliberate mode of creativity (Dietrich, 2019).

### Enriched Physical Education Effects on Motor Creativity: Direct and Mediated Paths

The mediation analysis provided some evidence on whether motor coordination and inhibition, as hypothesized by Richard et al. (2018a), and divergent creative thinking, as hypothesized by Bournelli and Mountakis (2008), are mechanisms that underlie the efficacy of motor creativity training programs. Although the specialist-led enriched PE caused significantly larger gains than generalist-led conventional PE in inhibition and in some facets of motor coordination and creative thinking, the extent to which these motor and cognitive skills improved did not explain the gains in motor fluency, flexibility, and originality, as indicated by the absence of a mediation path between pre-post delta scores. In the ecological school context, several influential and co-varying factors may have impeded to detect significant relations between intervention-related gains. Mediating mechanisms were found after the intervention, but not before it. Thus, whatever the size of the intervention-related gains, the enriched PE seems to align motor creativity to the level of specific prerequisites, likely rendering children capable to capitalize on these latter for moving creatively.

The hypothesis on the role of motor coordination was confirmed, as it partially explained the improved fluency, flexibility, and originality in moving after the intervention (Figures 3A–C). This suggests that children exposed to the enriched PE were able to use improved motor skills (or less impaired motor skills for those with atypical motor development at baseline) to explore and produce multiple, diversified and original movement solutions. Indeed, one of the cornerstones of the enriched PE was that the motor skills learnt were not conceived as the endpoint of learning but as new tools to extend, with non-linear pedagogy, the range of opportunities to explore and find new solutions (Adolph and Hoch, 2019). The constraints were manipulated to balance the extent to which the solution space was explored and the likelihood that children would vary either between different coordination categories or within one category (Hristovski et al., 2011).

Our results did not support the hypothesis of a mediating mechanism by creative thinking for any dimension of motor creativity. This fits with the emerging view that creative actions may not be the enactment of previously generated creative ideas, but are rather prompted by the affordances in the environment and their purposeful manipulation (Orth et al., 2017). This view has large similarity with Dietrich’s definition of flow mode of creativity, whose essence is proposed to be the perception-action coupling without any conscious control and creative thinking effort. In Dietrich’s words (2019, p. 4), “*The importance of a skilled movement sequence as a defining feature of the flow mode* (of processing in creativity) *cannot be overstated*,” as the motor system is deeply involved in creative thinking (Matheson and Kenett, 2020). To become fluent and original in moving, children seem not to require an enhanced efficiency of those cognitive processes that allow them to think out of the box. Rather, their ability to detect and exploit affordances, likely fostered by PE enrichment, may have translated into an ability to explore the ‘infinity’ within the box, that is, the variability potential of alternative ways to control a same coordination, without necessarily switching to new movement categories.

Instead, the improved ability to switch between movement coordination categories (i.e., motor flexibility) observed after the intervention was jointly explained by motor proficiency and inhibition (Figure 3B). The role of inhibition, which at a first sight seems to ground motor creativity in a cognitive framework has instead the potential to bridge the arguments on the emergence of motor creativity provided in the framework of Ecological Dynamics. Its non-linear pedagogical approach is assumed to facilitate the emergence of new functional patterns of motor coordination and control, because it maintains the perceptual-motor system in a region between stability and instability (meta-stable region; Hristovski et al., 2011; Torrents et al., 2021b). Speculatively, the ability to inhibit well-learnt, more common and therefore stable coordination patterns might help maintain the perceptual-motor system in this region of temporarily stable motor solutions which emerge based on actually selected affordances. Later on along the creative process, inhibition might come into play to enable a deliberate mode of processing and selection of creative behaviors (Cheng et al., 2016). Therefore, not inhibition *per se*, but an adaptive engagement of inhibition may matter (Benedek et al., 2012).

### Limitations

This study is not without limitations. Teachers and children could not be blinded; the higher session enjoyment in the enriched PE group might have been coupled with a tendency toward higher engagement. Nevertheless, adding the enjoyment as a covariate to the analysis did not alter the pattern of intervention effects. The intervention outcomes cannot be univocally attributed to the features of the enriched PE intervention, since this latter was delivered by specialists whereas the conventional PE was delivered by generalist classroom teachers. However, the qualitative analysis of teaching behaviors suggested the fidelity of the specialists’ teaching strategies to the targeted type of intervention and its likely contribution to the observed benefits. Moreover, the present study may have been underpowered to detect whether males and females are differently responsive to motor creativity interventions. Since evidence on sex differences in children’s motor creativity is scarce and mixed (Tocci et al., 2004; Ouhassine et al., 2020), future studies are warranted. A further limitation regards the low generalizability to school contexts in countries, which have jurisdictions with PE specialist teachers in every primary school. Furthermore, this study did not assess maintenance of the obtained improvements. Although a mediating role of the overall motor impairment score was found, the discriminative power of the M-ABC as a measure of motor coordination in children without DCD was suboptimal, as this tool is better suited to detect differences between typical and atypical motor development. The absence of mediating effects by working memory and those of inhibition being limited to only one facet of motor creativity might depend on the fact that executive functions were investigated with a task that taps them in decontextualized and affectively neutral conditions (i.e., ‘cool’ executive function) not comparable to an emotionally laden creative process in the motor domain. This raises the issue of how valid is, from an ecological perspective, a narrowly framed measurement of cognitive functions that consistently exhibit a narrow transfer (Kassai et al., 2019) to detect the multi-domain effects of holistic and hybridized pedagogical models (González-Víllora et al., 2018).

### Conclusions: Giving Ideas Some Legs or Legs Some Ideas?

The title provocatively asked whether children exposed to enriched PA learn how to give their ideas some legs, or how to give their legs some ideas. ‘Giving ideas some legs’ means ‘embodying’ creative ideas in pertinent and meaningful actions; ‘giving legs some ideas’ means, conversely, ‘enactive’ creativity that emerges through the intertwined processes of perceiving and acting (Malinin, 2019). Our study has mainly provided evidence in favor of enactive creativity promoted by the exposure to variation in constraints and supported by improved motor coordination (i.e., the sensorimotor ‘flow’ mode of creativity), but also some nuanced indication in support of embodied creativity through cognitive inhibition that likely enables to reject common or task-inappropriate movement categories and select novel ones (i.e., the ‘deliberate’ mode of creativity). This limited evidence for an involvement of ‘cool’ executive functions calls for research that assesses the role of ‘hot’ executive functions with affective aspects as those related to risk taking in decision making (Zelazo and Carlson, 2012). Cognitive functions that contribute to motion with e-motion may better reflect the motivational salience of the motor creativity context (Rudd et al., 2020) and are inherent in a recent overarching model of how enriched PA may enhance the creative potential (Richard et al., 2021). The need to address the salience of the context and the physical, cognitive, emotional and social facets of PA enrichment is emerging in movement sciences also in the first systematic attempt to identify contextualized mechanisms acting in the physical activity-cognition relation (Pesce et al., 2021b). These intriguing convergences may inspire future research that empirically develops at the intersection of ecological approaches to creativity, cognitive and movement sciences.

## Data Availability Statement

The datasets presented in this article are not readily available because of legal restrictions (i.e., national privacy legislation and inclusion, in the informed consent signed by parents/guardians, only of permission for communication of data in aggregated form). Data for secondary analyses (e.g., meta-analyses) can be rendered available by the corresponding author to individual researchers upon request. Requests to access the datasets should be directed to CP, caterina.pesce@uniroma4.it.

## Ethics Statement

The studies involving human participants were reviewed and approved by Ethics Committee of the “Umberto I” hospital of the First Rome University (Ref. No 2950). Written informed consent to participate in this study was provided by the participants’ legal guardian/next of kin.

## Author Contributions

CP, NT, and PS: conceptualization and methodology. EM and MM: validation. CP, EM, and MS: formal analysis. IM: investigation. NT, PS, and IM: data curation. CP: writing—original draft preparation and review, project administration, and funding acquisition. NT, PS, EM, MM, and MS: writing—review and editing for important intellectual content. CP and IM: supervision. All authors have read and agreed to the published version of the manuscript.

## Conflict of Interest

CP and IM received honoraria from Soremartec for developing and monitoring the enriched PE program and for training teachers. NT and PS received honoraria for training teachers. However, their role in the research project granted by the corporate has not influenced the outcomes reported in this article. EM, MM, and MS had no involvement in securing the funding for this research and received no financial support for the research, authorship, and/or publication of this article. The funders had no role in the design of the study; in the collection, analyses, or interpretation of data; in the writing of the manuscript, or in the decision to publish the results.

## Publisher’s Note

All claims expressed in this article are solely those of the authors and do not necessarily represent those of their affiliated organizations, or those of the publisher, the editors and the reviewers. Any product that may be evaluated in this article, or claim that may be made by its manufacturer, is not guaranteed or endorsed by the publisher.

## Supplementary Material

Supplementary Data Sheet 1 ‘Joy of Moving’ Sample Games.

Supplementary Data Sheet 2 Information on assessment tasks, validity and reliability.

## References

[B1] Aadland K. N. Ommundsen Y. Anderssen S. A. Brønnick K. S. Moe V. F. Resaland G. K. (2019). Effects of the Active Smarter Kids (ASK) physical activity school-based intervention on executive functions: a cluster-randomized controlled trial. *Scand. J. Educ. Res.* 63 214–228. 10.1080/00313831.2017.1336477

[B2] Adolph K. E. Hoch J. E. (2019). Motor development: embodied, embedded, enculturated, and enabling. *Annu. Rev. Psychol.* 70 141–164. 10.1146/annurev-psych-010418-102836 30256718 PMC6320716

[B3] Audiffren M. Tomporowski P. D. Zagrodnik J. (2009). Acute aerobic exercise and information processing: modulation of executive control in a Random Number Generation task. *Acta Psychol.* 132 85–95. 10.1016/j.actpsy.2009.06.008 19632661

[B4] Bailey R. Hillman C. Arent S. Petitpas A. (2013). Physical activity: an underestimated investment in human capital? *J. Phys. Act. Health* 10 289–308. 10.1123/jpah.10.3.289 23620387

[B5] Bardid F. Utesch T. (2018). “Motor competence,” in *Dictionary of Sport Psychology*, eds Hackfort S. R. Strauss B. (Amsterdam: Elsevier), 336.

[B6] Barnett L. M. Lai S. K. Veldman S. L. C. Hardy L. L. Cliff D. P. Morgan P. J. (2016). Correlates of gross motor competence in children and adolescents: a systematic review and meta-analysis. *Sports Med.* 46 1663–1688. 10.1007/s40279-016-0495-z 26894274 PMC5055571

[B7] Barnett L. M. Webster E. K. Hulteen R. M. De Meester A. Valentini N. C. Lenoir M. (2021). Through the looking glass: a systematic review of longitudinal evidence, providing new insight for motor competence and health. *Sports Med.* [Epub Online ahead of print]. 10.1007/s40279-021-01516-8 34463945 PMC8938405

[B8] Benedek M. Franz F. Heene M. Neubauer A. C. (2012). Differential effects of cognitive inhibition and intelligence on creativity. *Pers. Individ. Differ.* 53 480–485. 10.1016/j.paid.2012.04.014 22945970 PMC3387381

[B9] Bertsch J. (1983). *Le créativitè motrice. Son evaluation et son optimisation dans la pédagogie des situations motrices a l’ecole – Manuel de tests [Motor Creativity. Evaluation and Optimization in the Pedagogy of Physical Education – Test Manual].* Paris: INSEP.

[B10] Bournelli P. (1998). The development of motor creativity in elementary school children through a specific physical education program. *J. Biol. Exerc.* 3 68–82.

[B11] Bournelli P. Mountakis C. (2008). The development of motor creativity in elementary school children and its retention. *Creat. Res. J.* 20 72–80.

[B12] Cairney J. Dudley D. Kwan M. Bulten R. Kriellaars D. (2019). Physical literacy, physical activity and health: toward an evidence-informed conceptual model. *Sports Med.* 49 371–383. 10.1007/s40279-019-01063-3 30747375

[B13] Chatoupis C. (2013). Young children’s divergent movement ability: a study revisited. *Early Child Dev. Care* 183 92–108. 10.1080/03004430.2012.655728

[B14] Cheng L. Hu W. Jia X. Runco M. A. (2016). The different role of cognitive inhibition in early versus late creative problem finding. *Psychol. Aesthet. Creat. Arts* 10 32–41. 10.1037/aca0000036

[B15] Cole T. J. Bellizzi M. C. Flegal K. M. Dietz W. H. (2000). Establishing a standard definition for child overweight and obesity worldwide: international survey. *BMJ* 120 1240–1243. 10.1136/bmj.320.7244.1240 PMC27365 10797032

[B16] Cramond B. Matthews-Morgan J. Bandalos D. Zuo L. (2005). A report on the 40-year follow-up of the Torrance tests of creative thinking: alive and well in the new millennium. *Gift. Child Q.* 49 283–291. 10.1177/001698620504900402

[B17] Croce R. V. Horvat M. McCarthy E. (2001). Reliability and concurrent validity of the movement assessment battery for children. *Percept. Mot. Skills* 93 275–280. 10.2466/pms.93.5.275-280 11693695

[B18] De Dreu C. K. Nijstad B. A. Baas M. Wolsink I. Roskes M. (2012). Working memory benefits creative insight, musical improvisation, and original ideation through maintained task-focused attention. *Pers. Soc. Psychol. Bull.* 38 656–669. 10.1177/0146167211435795 22301457

[B19] Di Cagno A. Crova C. Pesce C. (2006). Effects of educational rhythm-based learning on coordinative motor performance and sports enjoyment of male and female pupils. *J. Hum. Mov. Stud.* 51 143–165.

[B20] Diamond A. Ling D. S. (2020). “Review of the evidence on, and fundamental questions about efforts to improve executive functions, including working memory,” in *Cognitive and Working Memory Training: Perspectives from Psychology, Neuroscience, and Human Development*, eds Novick J. Bunting M. F. Dougherty M. R. Engle R. W. (New York: Oxford University Press), 143–431. 10.1093/oso/9780199974467.003.0008

[B21] Dietrich A. (2019). Types of creativity. *Psychon. Bull. Rev.* 26 1–12. 10.3758/s13423-018-1517-7 30128937

[B22] Eddy L. H. Bingham D. D. Crossley K. L. Shahid N. F. Ellingham-Khan M. Otteslev A. (2020). The validity and reliability of observational assessment tools available to measure fundamental movement skills in school-age children: a systematic review. *PLoS One* 15:e0237919. 10.1371/journal.pone.0237919 32841268 PMC7447071

[B23] Furley P. Memmert D. (2015). Creativity and working memory capacity in sports: working memory capacity is not a limiting factor in creative decision making amongst skilled performers. *Front. Psychol.* 10:115. 10.3389/fpsyg.2015.00115 25713552 PMC4322539

[B24] González-Víllora S. Evangelio C. Sierra-Díaz M. J. Fernández-Río J. (2018). Hybridizing pedagogical models: a systematic review. *Eur. Phys. Educ. Rev.* 25 1056–1074. 10.1177/1356336x18797363

[B25] Grammatikopoulos V. Gregoriadis A. Zachopoulou E. (2012). “Acknowledging the role of motor domain in creativity in early childhood education,” in *Contemporary Perspectives on Research in Creativity in Early Childhood Education*, ed. Saracho O. (Charlotte: Information Age Publishing), 161–178.

[B26] Hayes A. F. (2013). *Introduction to Mediation, Moderation, and Conditional Process Analysis: A Regression-Based Approach.* New York: Guilford Press.

[B27] Hedges L. V. Hedberg E. C. (2007). Intraclass correlation values for planning group randomized trials in education. *Educ. Eval. Policy Anal.* 29 60–87. 10.3102/0162373707299706

[B28] Henderson S. E. Sudgen D. A. (1992). *Movement Assessment Battery for Children [Italian Version: (2000). Movement ABC—Batteria per la Valutazione Motoria del Bambino, eds E. Mercuri and E. Mercuri (Firenze: Giunti O.S.)].* London: The Psychological Corporation.

[B29] Hristovski R. Davids K. Araujo D. Passos P. (2011). Constraints-induced emergence of functional novelty in complex neurobiological systems: a basis for creativity in sport. *Nonlinear Dynamics Psychol. Life Sci.* 15 175–206. 21382260

[B30] Huizinga M. Dolan C. V. Van der Molen M. W. (2006). Age-related change in executive function: developmental trends and a latent variable analysis. *Neuropsychologia* 44 2017–2036. 10.1016/j.neuropsychologia.2006.01.010 16527316

[B31] Hulteen R. M. Morgan P. J. Barnett L. M. Stodden D. F. Lubans D. R. (2018). Development of foundational movement skills: a conceptual model for physical activity across the lifespan. *Sports Med.* 48 1533–1540. 10.1007/s40279-018-0892-6 29524160

[B32] Jefferies P. (2020). Physical literacy and resilience: the role of positive challenges. *Sci. Bonheur* 5 11–26.

[B33] Jiménez-Díaz J. Chaves-Castro K. Salazar W. (2019). Effects of different movement programs on motor competence: a systematic review with meta-analysis. *J. Phys. Act. Health* 16 657–666. 10.1123/jpah.2018-0179 31319403

[B34] Kassai R. Futo J. Demetrovics Z. Takacs Z. K. (2019). A meta-analysis of the experimental evidence on the near- and far-transfer effects among children’s executive function skills. *Psychol. Bull.* 145 165–188. 10.1037/bul0000180 30652908

[B35] Kell H. J. Lubinski D. Benbow C. P. Steiger J. H. (2013). Creativity and technical innovation: spatial ability’s unique role. *Psychol. Sci.* 24 1831–1836. 10.1177/0956797613478615 23846718

[B36] Khalil R. Karim A. A. Kondinska A. Godde B. (2020). Effects of transcranial direct current stimulation of left and right inferior frontal gyrus on creative divergent thinking are moderated by changes in inhibition control. *Brain Struct. Funct.* 225 1691–1704. 10.1007/s00429-020-02081-y 32556475 PMC7321900

[B37] Kim K. H. (2006). Can we trust creativity tests? A review of the Torrance tests of creative thinking (TTCT). *Creat. Res. J.* 18 3–14.

[B38] Malinin L. H. (2019). How radical is embodied creativity? Implications of 4E Approaches for Creativity Research and Teaching. *Front. Psychol.* 10:2372. 10.3389/fpsyg.2019.02372 31695653 PMC6818493

[B39] Marinšek M. Lukman N. (2021). Teaching strategies for promoting motor creativity and motor skill proficiency in early childhood. *Econ. Res. Ekon. Istraz* 10.1080/1331677X.2021.1974306

[B40] Matheson H. E. Kenett Y. N. (2020). The role of the motor system in generating creative thoughts. *Neuroimage* 213:116697. 10.1016/j.neuroimage.2020.116697 32142883

[B41] Memmert D. (2011). “Sports and creativity,” in *Encyclopedia of Creativity*, 2nd Edn, Vol. 2 eds Runco M. A. Pritzker S. R. (San Diego: Academic Press), 373–378. 10.1016/b978-0-12-375038-9.00207-7

[B42] Milić N. S. (2014). The influence of motor experience on motor creativity (fluency) of preschool children. *Kinesiology* 46 81–86.

[B43] Miyake A. Friedman N. P. Emerson M. J. Witzki A. H. Howerter A. Wager T. D. (2000). The unity and diversity of executive functions and their contributions to complex “frontal lobe” tasks: a latent variable analysis. *Cogn. Psychol.* 41 49–100. 10.1006/cogp.1999.0734 10945922

[B44] Moraru A. Memmert D. van der Kamp J. (2016). Motor creativity: the roles of attention breadth and working memory in a divergent doing task. *J. Cogn. Psychol.* 28 856–867. 10.1080/20445911.2016.1201084

[B45] Mosston M. Ashworth S. (2008). *Teaching Physical Education.* Available Online at: https://spectrumofteachingstyles.org/assets/files/book/Teaching_Physical_Edu_1st_Online.pdf (accessed October 29, 2021).

[B46] Ng J. L. Button C. (2018). Reconsidering the fundamental movement skills construct: implications for assessment. *Mov. Sport Sci.* 102 19–29. 10.1051/sm/2018025

[B47] Orth D. van der Kamp J. Memmert D. Savelsbergh G. J. P. (2017). Creative motor actions as emerging from movement variability. *Front. Psychol.* 8:1903. 10.3389/fpsyg.2017.01903 29163284 PMC5671646

[B48] Ouhassine I. Seghir N. E. Guerrach L. Cheikh Y. Boufadene O. Chenouf K. (2020). Male and female differences in motor creativity at the age of 6 and 7 years. *Int. J. Appl. Exerc. Physiol.* 9 134–139.

[B49] Pesce C. Croce R. Ben-Soussan T. D. Vazou S. McCullick B. Tomporowski P. D. (2019). Variability of practice as an interface between motor and cognitive development. *Int. J. Sport Exerc. Psychol.* 17 133–152. 10.1080/1612197X.2016.1223421

[B50] Pesce C. Marchetti R. Motta A. Bellucci M. (2016a). *Joy of Moving. MindMovers* & *ImmaginAction - Playing with Variability to Promote Motor, Cognitive and Citizenship Development.* Perugia: Calzetti-Mariucci.

[B51] Pesce C. Masci C. Marchetti R. Vazou S. Sääkslahti A. Tomporowski P. D. (2016b). Deliberate play and preparation jointly benefit motor and cognitive development: mediated and moderated effects. *Front. Psychol.* 7:349. 10.3389/fpsyg.2016.00349 27014155 PMC4786558

[B52] Pesce C. Stodden D. F. Lakes K. D. (2021a). Physical activity “enrichment”: a joint focus on motor competence, hot and cool executive functions. *Front. Psychol.* 12:658667. 10.3389/fpsyg.2021.658667 33767654 PMC7985325

[B53] Pesce C. Vazou S. Benzing V. Alvarez-Bueno C. Anzeneder S. Mavilidi M. (2021b). Effects of chronic physical activity on cognition across the lifespan: a systematic meta-review of randomized controlled trials and realist synthesis of contextualized mechanisms. *Int. Rev. Sport Exerc. Psychol.* [Epub ahead of print]. 10.1080/1750984X.2021.1929404

[B54] Radel R. Davranche K. Fournier M. Dietrich A. (2015). The role of (dis)inhibition in creativity: decreased inhibition improves idea generation. *Cognition* 134 110–120. 10.1016/j.cognition.2014.09.001 25460384

[B55] Rasmussen L. J. T. Østergaard L. D. (2016). The creative soccer platform: new strategies for stimulating creativity in organized youth soccer practice. *J. Phys. Educ. Recreat. Dance* 87 9–19. 10.1080/07303084.2016.1202799

[B56] Resaland G. K. Moe V. F. Aadland E. Steene-Johannessen J. Glosvik Ø Andersen J. R. (2015). Active Smarter Kids (ASK): rationale and design of a cluster-randomized controlled trial investigating the effects of daily physical activity on children’s academic performance and risk factors for non-communicable diseases. *BMC Public Health* 15:709. 10.1186/s12889-015-2049-y 26215478 PMC4517398

[B57] Richard V. Holder D. Cairney J. (2021). Creativity in motion: examining the creative potential system and enriched movement activities as a way to ignite it. *Front. Psychol.* 12:690710. 10.3389/fpsyg.2021.690710 34659006 PMC8514639

[B58] Richard V. Lebeau J. C. Becker F. Boiangin N. Tenenbaum G. (2018a). Developing cognitive and motor creativity in children through an exercise program using nonlinear pedagogy principles. *Creat. Res. J.* 30 391–401. 10.1080/10400419.2018.1530913

[B59] Richard V. Lebeau J. C. Becker F. Inglis E. R. Tenenbaum G. (2018b). Do more creative people adapt better? An investigation into the association between creativity and adaptation. *Psychol. Sport Exerc.* 38 80–89. 10.1016/j.psychsport.2018.06.001

[B60] Rink J. E. (2006). *Teaching Physical Education for Learning*, 5th Edn. Boston: McGraw Hill.

[B61] Rudd J. R. Foulkes J. D. O’Sullivan M. Woods C. T. (2021a). “A ‘fundamental’ myth of movement with a ‘functional’ solution,” in *Myths of Sport Coaching*, eds Whitehead A. Coe J. (Yorkshire: Sequoia Books).

[B62] Rudd J. R. Woods C. Correia V. Seifert L. Davids K. (2021b). An ecological dynamics conceptualisation of physical ‘education’: where we have been and where we could go next. *Phys. Educ. Sport Pedagogy* 26 293–306. 10.1080/17408989.2021.1886271

[B63] Rudd J. R. Pesce C. Strafford B. W. Davids K. (2020). Physical literacy – A journey of individual enrichment: an ecological dynamics rationale for enhancing performance and physical activity in all. *Front. Psychol.* 11:1904. 10.3389/fpsyg.2020.01904 32849114 PMC7399225

[B64] Santos S. JimeÂnez S. Sampaio J. Leite N. (2017). Effects of the Skills4Genius sports-based training program in creative behavior. *PLoS One* 12:e0172520. 10.1371/journal.pone.0172520 28231260 PMC5322953

[B65] Santos S. Monteiro D. (2021). Uncovering the role of motor performance and creative thinking on sports creativity in primary school-aged children. *Creat. Res. J.* 33 1–15. 10.1080/10400419.2020.1843125

[B66] Schmidt R. A. Lee T. D. Winstein C. Wulf G. Zelaznik H. N. (2011). *Motor Control and Learning: A Behavioural Emphasis*, 6th Edn. Champaign: Human Kinetics.

[B67] Schulz J. Henderson S. E. Sugden D. A. Barnett A. L. (2011). Structural validity of the Movement ABC-2 test: factor structure comparisons across three age groups. *Res. Dev. Disabil.* 32 1361–1369. 10.1016/j.ridd.2011.01.032 21330102

[B68] Scibinetti P. (2019). *Creatività motoria. Come Svilupparla in età Evolutiva e Anziana [Motor Creativity. How to Promote it During Development and Aging].* Perugia: Calzetti-Mariucci.

[B69] Scibinetti P. Tocci N. Pesce C. (2011). Motor creativity and creative thinking in children: the diverging role of inhibition. *Creat. Res. J.* 23 262–272. 10.1080/10400419.2011.595993

[B70] Stodden D. Lakes K. Côté J. Aadland E. Benzing V. Brian A. (2021). Exploration: an overarching focus for holistic development. *Braz. J. Mot. Behav.* 15 301–320. 10.20338/bjmb.v15i5.254

[B71] Thomaidou C. Konstantinidou E. Venetsanou F. (2021). Effects of an eight-week creative dance and movement program on motor creativity and motor competence of preschoolers. *J. Phys. Educ. Sport* 21 3268–3277. 10.7752/jpes.2021.s6445 33809896

[B72] Tocci N. Scibinetti P. (2007). Essere creativi è utile [Being creative is useful]. *SDS Riv. Cult. Sportiva* 72 53–61.

[B73] Tocci N. Scibinetti P. Zelli A. (2004). Age and gender differences in motor creativity among Italian elementary school children. *J. Hum. Mov. Stud.* 46 89–104. 10.31470/2308-5126-2019-41-1-89-98

[B74] Tomporowski P. D. McCullick B. Pendelton D. M. Pesce C. (2015a). Exercise and children’s cognition: the role of exercise characteristics and a place for metacognition. *J. Sport Health Sci.* 4 47–55. 10.1016/j.jshs.2014.09.003

[B75] Tomporowski P. D. McCullick B. A. Pesce C. (2015b). *Enhancing Children’s Cognition with Physical Activity Games.* Champaign: Human Kinetics.

[B76] Tomporowski P. D. McCullick B. A. Horvat M. (2010). *Role of Contextual Interference and Mental Engagement on Learning.* New York: Nova Science Publishers, Inc.

[B77] Torrance E. P. (1988). *Guidelines for Administration and Scoring/Comments on Using the Torrance Tests of Creative Thinking [Italian Version: (1989). Test di Pensiero Creativo. Firenze: Organizzazioni Speciali].* Bensenville, IL: Scholastic Testing Service Inc.

[B78] Torrance E. P. Ball O. E. (1984). *The Torrance Tests of Creative Thinking Streamlined (revised) Manual, Figural A and B.* Bensenville: Scholastic Testing Service, Inc.

[B79] Torrents C. Balagué N. Hristovski R. Almarcha M. Kelso J. A. S. (2021a). Metastable Coordination dynamics of collaborative creativity in educational settings. *Sustainability* 13:2696. 10.3390/su13052696

[B80] Torrents C. Balagué N. Ric Á. Hristovski R. (2021b). The motor creativity paradox: constraining to release degrees of freedom. *Psychol. Aesthet. Creat. Arts* 15 340–351. 10.1037/aca0000291

[B81] Torrents C. Ric Á. Hristovski R. (2015). Creativity and emergence of specific dance movements using instructional constraints. *Psychol. Aesthet. Creat. Arts* 9 65–74. 10.1037/a0038706

[B82] Towse J. N. McIachlan A. (1999). An exploration of random generation among children. *Br. J. Dev. Psychol.* 17 363–380. 10.1348/026151099165348

[B83] Towse J. N. Neil D. (1998). Analyzing human random generation behavior: a review of methods used and a computer program for describing performance. *Behav. Res. Meth. Instrum. Comput.* 30 583–591. 10.3758/BF03209475

[B84] van der Fels I. M. Te Wierike S. C. Hartman E. Elferink-Gemser M. T. Smith J. Visscher C. (2015). The relationship between motor skills and cognitive skills in 4-16 year old typically developing children: a systematic review. *J. Sci. Med. Sport* 18 697–703. 10.1016/j.jsams.2014.09.007 25311901

[B85] Veitch J. Salmon J. Ball K. (2009). The validity and reliability of an instrument to assess children’s outdoor play in various locations. *J. Sci. Med. Sport* 12 579–582. 10.1016/j.jsams.2008.09.001 19027361

[B86] Zelazo P. D. Carlson S. M. (2012). Hot and cool executive function in childhood and adolescence: development and plasticity. *Child Dev. Perspect.* 6 354–360. 10.1111/j.1750-8606.2012.00246.x

[B87] Zhang W. Sjoerds Z. Hommel B. (2020). Metacontrol of human creativity: the neurocognitive mechanisms of convergent and divergent thinking. *Neuroimage* 210:116572. 10.1016/j.neuroimage.2020.116572 31972282

