# Supplementary material for: Giving Ideas Some Legs or Legs Some Ideas? Children’s Motor Creativity Is Enhanced by Physical Activity Enrichment: Direct and Mediated Paths
Source: Front Psychol. 2022 Mar 10;13:806065. doi: 10.3389/fpsyg.2022.806065 (PMC8960453; doi:10.3389/fpsyg.2022.806065)
Supplement: Supplementary Data Sheet 1 — ‘Joy of Moving’ Sample Games. [file Data_Sheet_1.PDF]

Watch game videos online

Joy of moving

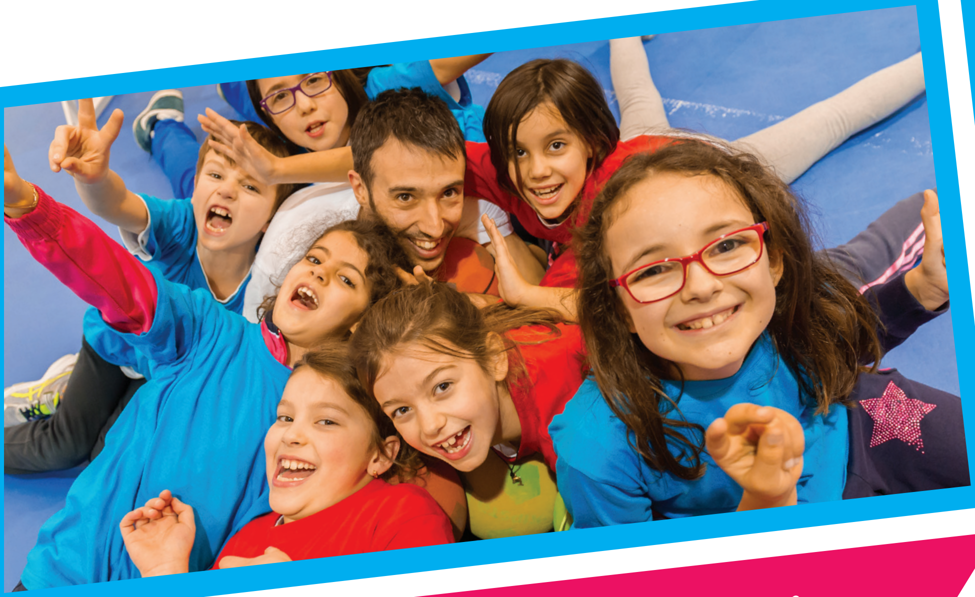

MindMovers & ImaginAction

Playing with variability
to promote motor, cognitive
and citizenship development

Edited by
**Caterina Pesce, Rosalba Marchetti,
Anna Motta, Mario Bellucci**

CALZETTI MARIUCCI
e d i t o r i

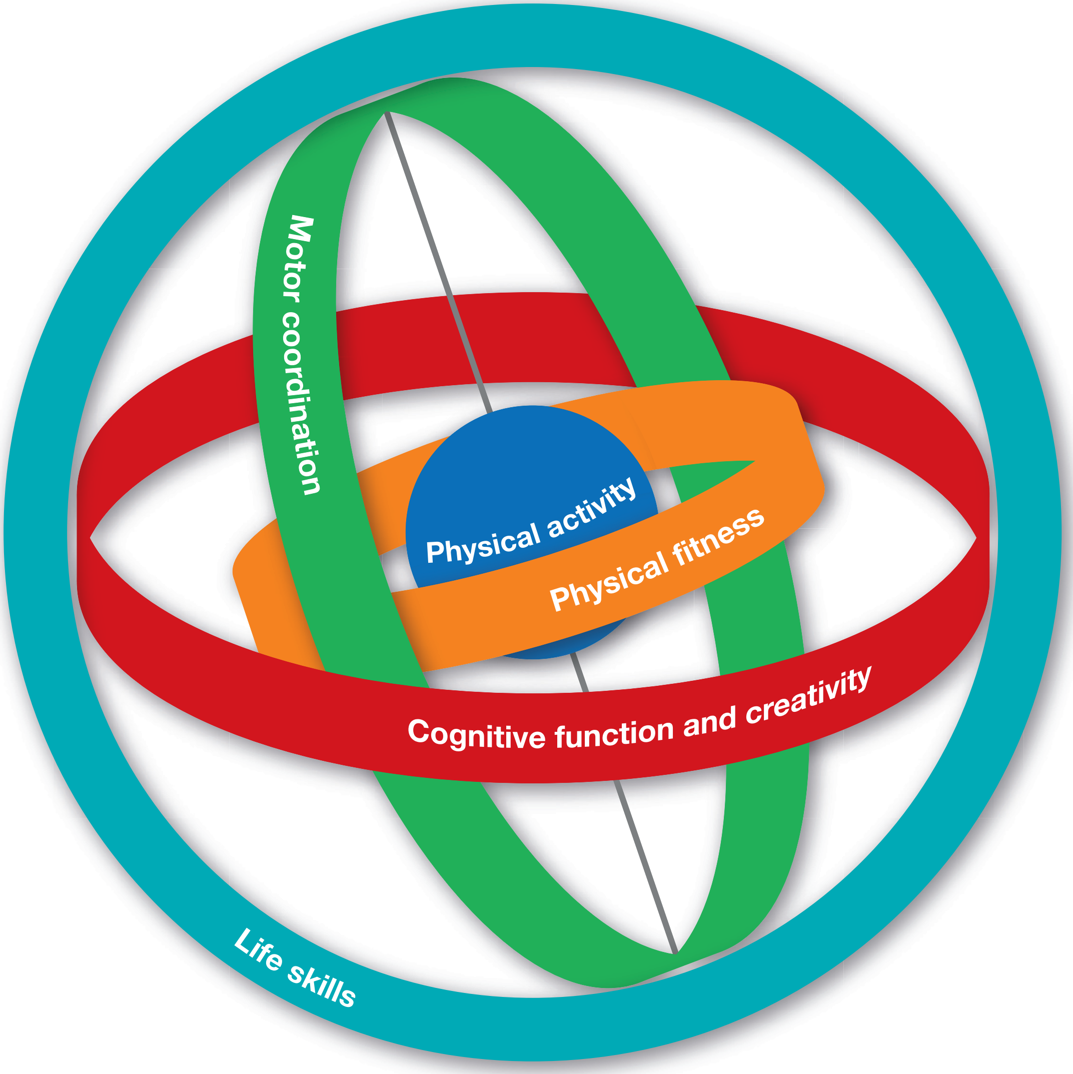
Motor coordination
Physical activity
Physical fitness
Cognitive function and creativity
Life skills

66

MAGNETS AND MECHANISMS

www.joyofmovinghandbook.com/en/games/66-magnets-mechanisms

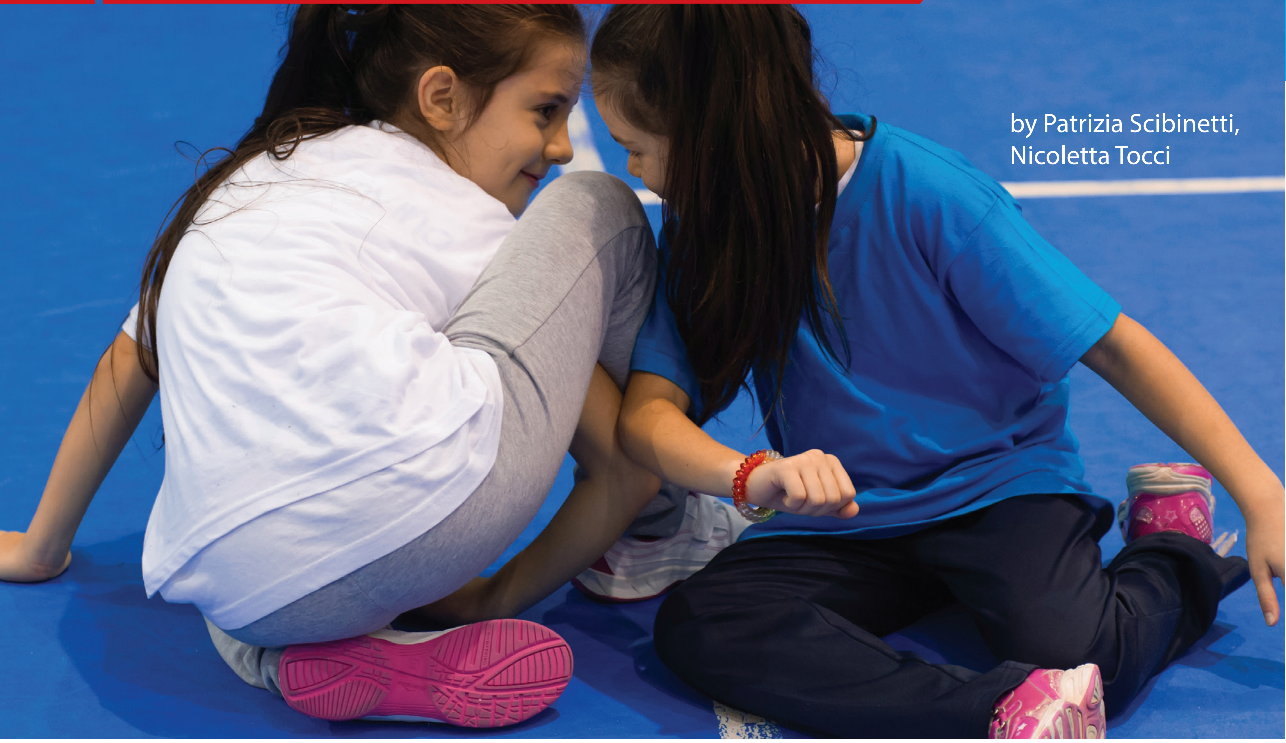

by Patrizia Scibinetti, Nicoletta Tocci

Age group

From **6** years upwards

What you need

Wide open space and music.

How to play

The children move about, touching the part of their body that the educator calls out, with that of the nearest playmate or playmates.

Possible variations

- With two/three parts of the body.
- With two or more playmates.
- Moving around the play area.

Before starting the game

Touch and move the body part called out by the educator, and repeat its name.
Feel how the joints move.
Experiment different positions (kneeling down, lying down, on all fours, etc.)

Aim of the game

The game aims at stimulating the perception and knowledge of different body parts. It also requires younger children to recognize, identify and name the different parts of the body. The game evolves with the subsequent construction of increasingly complex moving gears, putting different body segments in contact with those of fellow playmates. The aim is to construct a mechanism whose operation depends on the precise collaboration and interaction of each piece.

How to play – Instructions for use

The educator asks the children to move around freely in the play area, with music in the background to stimulate movement. He then stops the music and indicates a part of the body which, like a magnet, will have to be placed in contact with the same part of the closest playmate, staying still and in different positions.

The educator then indicates to the children-magnet two/three different parts of the body to be placed in contact with the closest playmate, staying still, in different positions.

How to develop the game
From repetition to change

A ***The gears***. The children move about freely and, when the music stops, they become gears that interlock, using the parts of the body indicated by educator:

- in small groups (3-5 children), choosing unusual, original positions;
- in pairs, in different positions without losing contact, moving freely;
- in small groups, in different positions without losing contact, moving freely;
- put two different parts of the body in contact with the closest playmate in different positions, continue moving without losing contact;
- put two different parts of the body in contact with closest playmates, continue moving in small groups, without losing contact and in different positions.

B ***From constructing a mechanism to building a futuristic car.***

As mentioned in the introduction, a variation of the game involves dividing the class into two groups. In complete autonomy, they will have to build the mechanics of a car of the future that moves through space. They do this by putting in contact the parts of the body that they consider appropriate to build the car. From the creative point of view, the difficulty arises from the fact that there are no actual prototypes for this in real life and there are infinite possibilities of contact between the various parts of the body. Furthermore, to make the object's movement as functional as possible, it will be necessary to modify the

parts of the body that come into contact during the action. There is no longer the constraint of the parts dictated by the educator, they can be changed at any time.

C ***The sticky ball***. Unlike previous experiences, contact between the various body parts comes about via a ball, placed between the pair's two body parts. The pair must move freely around the play area, assuming the various positions, without dropping the ball and following its natural movement, which depending on its form, will always position itself on or between different parts of the body. The use of the hands is forbidden. The goal is to experiment unusual movements and actions in pairs, so the ball never loses contact with the bodies.

D ***Thermal baths***. The mechanism is identical to the previous variation, but it is more difficult as it must be performed in a group of four with a single ball. Given the complexity of the task, it is best to start on the ground.

How to make the game work – Teaching strategies

With younger children, who do not yet know the parts of the body well, the educator indicates the part to be put in contact when she calls it out. Initially, she indicates the same body part for all the pairs/small groups, then she assigns different parts for each child (for example, in pairs: one child's shoulder in contact with the other's knee).

The objective being to produce unusual forms of contact and movements, the educator should try to indicate parts of the body that are distant from each other or on different sides of the body. Should the children's responses be stereotypical, the space where the task is performed can be restricted (for example, only on the ground or only in an upright position, and lastly with a continuous alternation of positions). No suggestions should be given: when the educator indicates several parts of the body, the children are free to choose whether or not to put in contact all the body parts required; then, when the educator does not specify several parts of the body to be put in contact, the children are free to choose which parts of the body to use.

Physical fitness objectives

Improving joint flexibility and muscle tone.

Motor coordination objectives

Improving the perception and knowledge of the different body parts. As construction of the mechanism gradually progresses, the task stimulates accurate motor control abilities (interlimb coordination). In relation to the parts to be kept in contact, the type of movements, passing from one position to another, this game stimulates balance and orientation ability. Moreover, given the constant need to change one's actions according to the playmate's movements, it serves to improve both accurate and, at certain times, fast perceptual-motor adaptation abilities (reaction and transformation).

Cognitive and creativity objectives

Improving motor creativity and creative thinking, through the free choice of alternative solutions, and the ability to inhibit routine and common responses (application of the **stop principle**[1]).

Life skills objectives

Intrapersonal - Interpersonal: improving self-regulation, cooperation and the ability to work in teams to build a mechanism whose operation depends on the interaction of each individual child. Stimulating the ability to pursue a common goal (**goal setting**[2], **decision making**[3]) and the ability to find practical and relevant solutions to emerging problems (**problem solving**[4]), which will only be valid if sharable through effective communication.

Keywords

1 - **Stop principle:** applied to tasks where an external stimulus indicates that the person must interrupt an action (e.g. the game 'One, two, three... Star!' analyzed in chapter 1).

2 - **Goal setting:** developing an action plan designed to motivate and guide a person or a group towards the achievement of a goal.

3 - **Decision making:** the ability to make effective decisions under time pressure, considering the objectives and the consequences of alternative actions.

4 - **Problem solving:** the ability to solve problems that arise in a relevant and efficient way.

63 A FRIEND IS A TREASURE

www.joyofmovinghandbook.com/en/games/63-friend-treasure

by Caterina Pesce, Claudia Crova

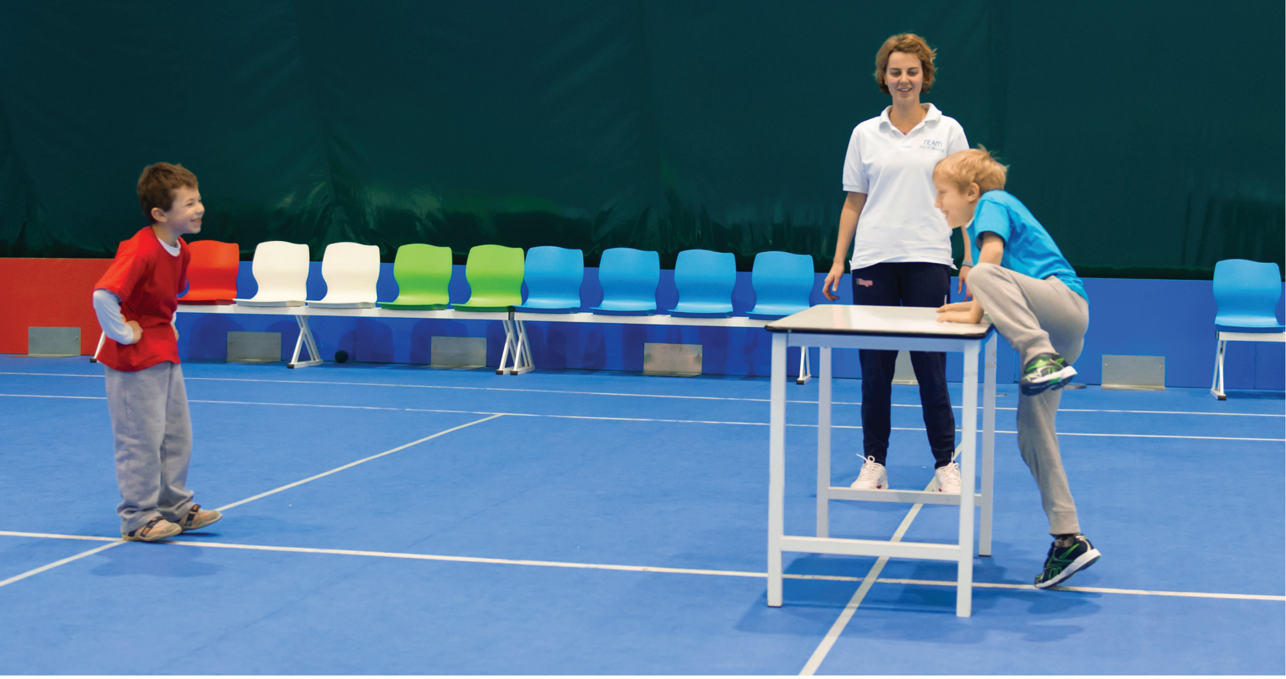

Age group

From **7** years upwards

What you need

One or more tables, crash mats.

How to play

In groups of 5-10, one child at a time must reach (reconcile with) a friend, getting past the table (obstacle) between them in various ways.

Possible variations

- Repeating playmates' movement with the addition of details (a sequence of clues).
- A sequence of clues in reverse order.
- The opposites game: children do the opposite actions to those of their playmates.
- Perform the sequence of movements backwards.
- In pairs.

Before starting the game

The children position the tables in the play area, putting crash mats under, in front of and behind the tables, to create a safe play environment.

Aim of the game

This game trains cognitive flexibility and memory. The game takes on a socio-affective connotation, as there is an obstacle (table) between the child and his friend that must be overcome so that they can hug each other.

How to play – Instructions for use

The children are divided into small groups of 5-10, each with one table, or they form a single subgroup with one table, while the other subgroups are engaged in parallel tasks that are easy to supervise, so the children are not standing in line idle, waiting for their turn. Half the group stands in a line on one side of the table, a few meters away from it, while one child stands on the other side of the table, quite near it, facing the rest of the group. The educator must encourage the children to empathize in this symbolic game, telling them that the first child in the line and the child across the table have quarreled. The quarrel represents an obstacle (the table) to their reconciliation, which will come about when the first child in line, who decides to put an end to this friction, has reached his friend by climbing over, under and over again the obstacle that separates them. Once the first child in line has achieved his goal and has reached and hugged his friend on the other side of the table, he takes his place, while the playmate who was behind the table crosses over and goes to the back of the line. The game continues until everyone has tried to get over the table at least once.

How to develop the game
From repetition to change

After all the group has experimented the two roles (reaching the friend and being the child who is reached and hugged), the children repeat the game respecting some limitations: to do this, they must make the effort to carefully observe and memorize each playmate's action.

A ***A hunt for clues***. The second child starts by making the same movements as the first, adding only one small detail, the clue (for example, banging her hands on the table). The playmate after her must look for the clue, repeat it and add another detail. In the end, the last child's movement will be very different from the first, since it will include all the clues created by each of the playmates gone before her.

B Repeat in reverse order the sequence of movements created in variation A, by gradually removing a detail to return to the original movement. Make sure that it is the exact same movement as the initial one.

C *The opposites games*. Each child must get over the obstacle in a totally different way from the previous child, by doing the opposite action: for example, if the first child passed under the table, the second child has to climb over it, and the third child will, in turn, choose an opposite action to that of the second, in other words, walking instead of running, and so on until the first child's movement has been transformed into something completely different.

D Repeat variation C backwards (the same procedure as variation B), gradually returning to the previous version.

E Repeat in pairs.

How to make the game work - Teaching strategies

As the variations introduce an element of increasing complexity, initially the child whose turn it is, can be asked to make a sound when inserting his new 'clue' into the sequence, so as to draw the attention of the playmate who will have to imitate him.

The educator can also organize groups in order to have a pair of 'assistants', whose job is to check the motor sequences should some playmates be unsure, especially when they have to perform the sequence backwards.

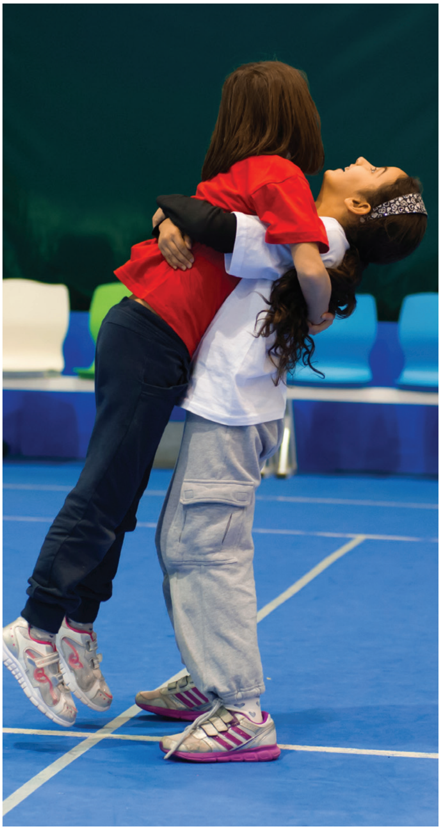

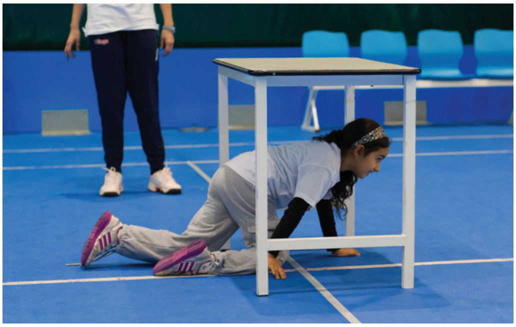

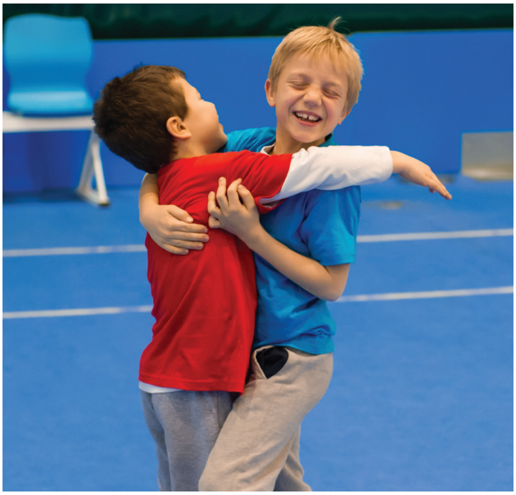

Getting inside the game

Physical fitness objectives

Improving strength, speed and joint flexibility.

Motor coordination objectives

Improving agility, accurate motor control abilities and accurate perceptual-motor adaptation abilities, as well as spatio-temporal orientation ability.

Cognitive and creativity objectives

Improving the ability to inhibit routine behaviors to respect the limitations given, adopting new and diversified solutions (creativity). Improving the management of attentional resources, for attention to be focused on the new characterizing elements, gradually added to the continued variations of the sequences for getting over the table.

Life skills objectives

Intrapersonal: improving memory and problem solving skills in a divergent and pertinent way.

Interpersonal: enhancing empathy and relational and cooperative skills, particularly in variation E.

78 A FRIEND IS A TREASURE

www.joyofmovinghandbook.com/en/games/78-friend-treasure

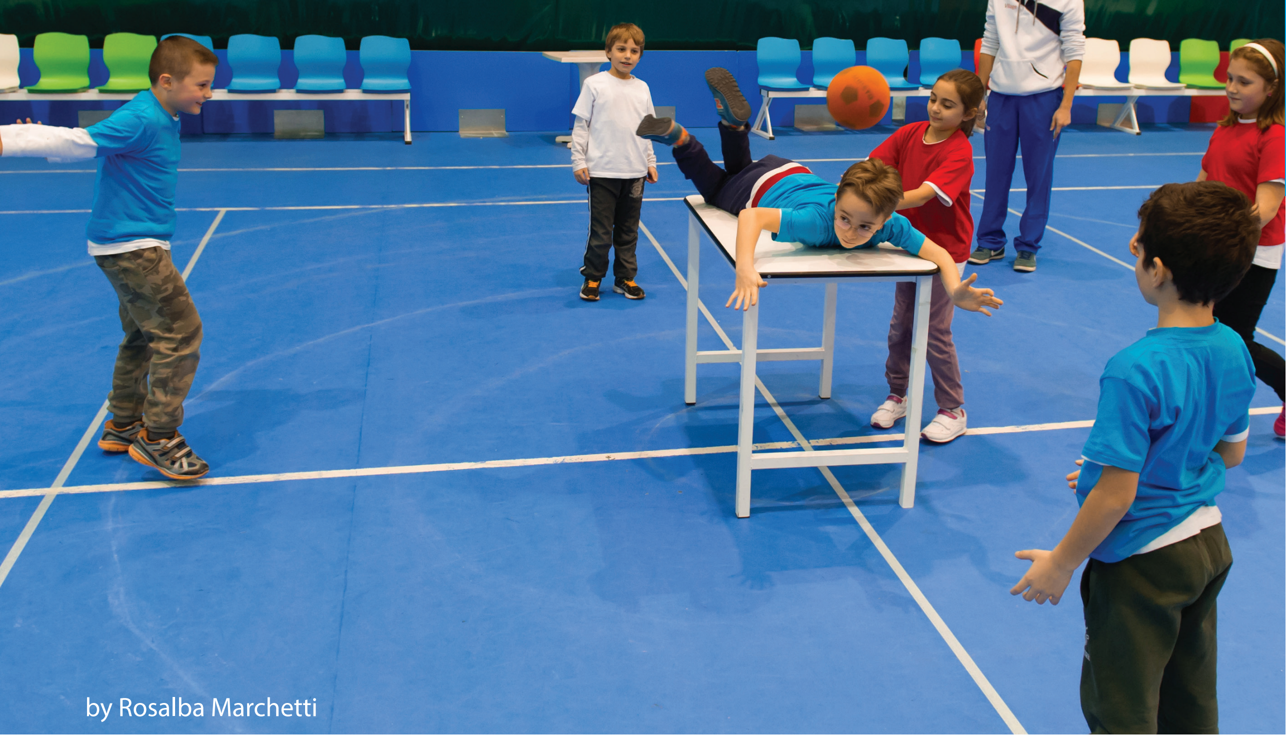

by Rosalba Marchetti

Age group

From **7** years upwards

What you need

One or more tables, crash mats, big and small foam balls.

How to play

In groups of 5-10, one child at a time must reach (reconcile with) a friend, getting past the table (obstacle) between them in various ways.

Possible variations

- With restrictions on how to perform and help from playmates.
- With interference from disturbers.
- With interference from disturbers and defense by teammates.

Before starting the game

The children position the tables in the play area, putting crash mats under, in front of and behind the tables to create a safe play environment.

Aim of the game

This game, already presented in chapter 9.1.2 dedicated to cognitive functions, more specifically in the section on cognitive flexibility, is oriented in this version towards the development of problem solving skills. In order for this motor 'problem solving' game to facilitate the transfer of this skill from the game to other life contexts, a child's everyday life situation is simulated (a metaphor of decision-making in a social situation): there is an obstacle (table) between the child and the friend that must be got past so the two can meet and make peace. The game, in this version, stimulates the search for new strategic solutions, introducing cooperation and defense against disruptive actions.

How to play - Instructions for use

The children are divided into small groups of 5-10, each with one table, or they form a single subgroup with one table, while the other subgroups are engaged in parallel tasks that are easy to supervise, so the children are not standing in line idle, waiting for their turn. Half the group stands in a line on one side of the table, a few meters away from it, while one child stands on the other side of the table, quite near it, facing the rest of the group. The educator must encourage the children to empathize in this symbolic game, telling them that the first child in the line and the child across the table have quarreled. The quarrel represents an obstacle (the table) to their reconciliation, which will come about when the first child in line, who decides to put an end to this friction, has reached his friend by climbing over, under and over again the obstacle that separates them. Once the first child in line has achieved his goal and has reached and hugged his friend on the other side of the table, he takes his place, while the playmate who was behind the table crosses over and goes to the back of the line. The game continues until everyone has tried to solve the problem at least once.

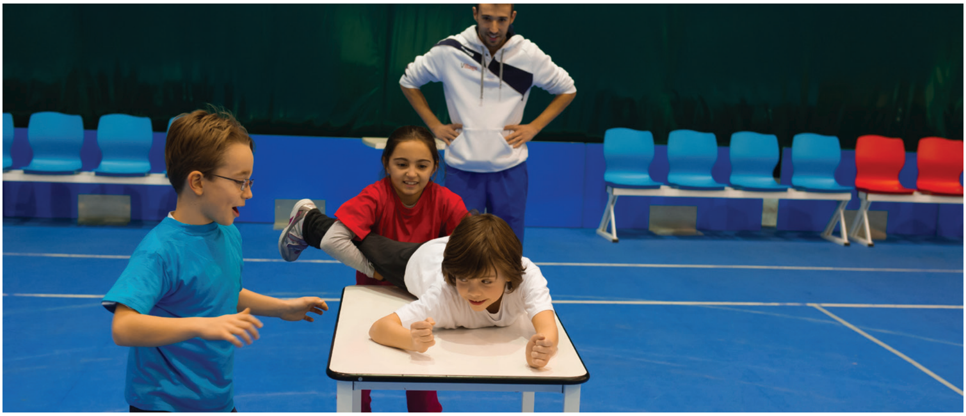

How to develop the game
From repetition to change

Restrict the ways in which the children can get past the table:

A ***Supportive relationships***. Pass over-under-over the table without touching the ground under the table with any part of the body. How can it be done? Ask teammates for help in finding cooperative strategies:
- first the teammates are allowed to touch both their playmate and the table;
- then they can only touch their playmate, but not the table.

B ***Defensive cooperation.*** Some of the children of a second sub-group, playing in parallel, make a circle around the first group's table and act as 'disturbers'. For example, playing dodgeball, passing a foam ball over the table or setting as in volleyball, depending on age and skill: after 7 passes (or sets) performed without dropping the ball, they can spike the ball in order to hit the person trying to get past the table and they must succeed before the child is able to get over-under-over the table. There are two variations:
- the teammates of the child getting past the table can 'block' the ball, to prevent it hitting him;
- the teammates cannot block the ball.

How to make the game work – Teaching strategies

To develop the ability to solve problems, the educator can make the task more complex and stimulate the search for more creative divergent solutions, by imposing some restrictions on how to perform it, or she can guide the children in the direction of tactical creativity, even setting some time constraints: she continually creates 'problems', gradually introducing new restrictions and stimulating different ways to comply with the instructions given. Example:

- a time limit is set for the first round (for example, all the children count up to ten or from ten down to zero,) within which the child has to reach his friend;
- in the second round the child has to bring a gift (this may be a small piece of equipment) to his friend;
- in the third round, the time constraint is removed, but the child must go over and under the table, touching it with any part of the body except his feet;
- in the fourth round, tell the children not to place their hands or feet on the table; etc.

With younger children, the motor proposals can be conveyed more effectively through fabulation. The educator tell the children, for example, to imagine that they are small animals fleeing from a large predator and that, to get away without being caught, they will have to be careful not to leave any footprints or fingerprints, justifying why they cannot place their hands or feet on or under the table.

In educating life skills it is always necessary to introduce pauses for reflection. At the end of the game, using an interactive method, the children should be made aware that: 1) if there is time pressure, or if someone tries to hinder the solution process (the educator counting to 10 or disturbers playing dodgeball), they may fall back on the most obvious and simple solutions of the task, which are not necessarily the best; 2) if the task becomes more difficult, it is a good idea to resort to the support and cooperation of others; 3) focusing reflection on the symbolic situation of the obstacle between two friends, it is good to make them aware of the fact that the friend who helps solve the problem can do so either by coming into direct contact with the problem itself (moving the table to help the friend get past), but also by supporting the friend without being directly involved in the problem (that is, symbolically, without touching the table); 4) the cooperation of individuals that help is not just necessary to solve the task in a pertinent way when it becomes more difficult (cooperative solution), but also to block the disturbing elements; 5) the probability of success in solving the task, in the presence of disturbing elements, increases when friendly forces actively help 'block' them.

Getting inside the game

Physical fitness objectives

Improving strength, speed and joint flexibility.

Motor coordination objectives

Improving agility, accurate and fast motor control abilities and fast and accurate perceptual-motor adaptation abilities (spatial-temporal orientation in dodging the balls and in aiming for the playmates getting past the table).

Cognitive and creativity objectives

Improving the ability to inhibit routine behaviors to respect the given constraints, adopting new and different solutions, even under time pressure, in order to deal with the disturbers' actions (tactical creativity).

Life skills objectives

Intrapersonal: improving problem solving skills in a divergent way and the ability to verify pertinence and the possible consequences of alternative actions.

Interpersonal: enhancing empathy and relational and cooperative skills.
