# Supplementary material for: Giving Ideas Some Legs or Legs Some Ideas? Children’s Motor Creativity Is Enhanced by Physical Activity Enrichment: Direct and Mediated Paths
Source: Front Psychol. 2022 Mar 10;13:806065. doi: 10.3389/fpsyg.2022.806065 (PMC8960453; doi:10.3389/fpsyg.2022.806065)
Supplement: Supplementary Data Sheet 2 — Information on assessment tasks, validity and reliability. [file Data_Sheet_2.PDF]

Supplementary material 2: Information on assessment tasks, validity and reliability

Bertsch's test of motor creativity

The Bertsch's test (1983) is validated for primary school children. The test is available in two separate versions (form A and B). For this study, we used the Bertsch's test form B. Flexibility and originality were coded and scored based on data obtained by Bertsch (1983) with his initial observation of nearly 7,000 different behaviours in children.

The test has an acceptable convergent validity (Bertsch, 1983), as revealed by mostly moderate correlations (r = .30–.65) with Wrick's (1968) Motor Creativity Test scores, with strong correlation (r = .65) reached by the total motor creativity score of Bertsch's test form B. The test also has an acceptable reliability (Bertsch, 1983), as revealed by mostly moderate day-to-day test-retest correlations across tasks and creativity dimensions (r = .31–.58, except a lower [.22] correlation of flexibility scores in the ball task). Lastly, the test has an acceptable internal consistency, as indexed by the Cronbach's alpha coefficient across the four tasks (α = .76; Scibinetti et al., 2011).

Movement Assessment Battery for Children

Manual dexterity

First task: 'posting coins', 'placing pegs' or 'shifting pegs by rows' (for 5-6, 7-8 or 9-10 year-old children, respectively). The child must drop coins through the slot in a bank box, or place 12 plastic pegs in all holes on a board, or move pegs from a given row to another, respectively, one at a time as quickly as possible. The seconds taken to complete each task were measured.

Second task: 'threading beads', 'threading lace' or 'threading nuts on bolt' (for 5-6, 7-8 or 9-10 year-old children, respectively). The child must thread beads through a lace, or thread a lace back and forth through the holes in a lacing board, or screw nuts down a bolt, respectively, one at a time as quickly as possible. The seconds taken to complete each task were measured.

Third task: 'bicycle trail' or 'flower trail' (for 5-6 or 7-10 year-old children, respectively). The child must draw with the preferred hand one continuous line following the flower trail on a record form without crossing its boundaries. The number of times the drawn lines moved outside a boundary (i.e., errors) was recorded.

Aiming and catching skills.

First task: 'catching bean bag', 'one-hand bounce and catch' or 'two-hand catch' (for 5-6, 7-8 or 9-10 year-old children, respectively). The child must catch a bean bag tossed by the experimenter, or bounce a tennis ball on the floor and catch it with the same hand, or throw a tennis ball at the wall form behind a marked line and catch it at the return with both hands, respectively. The number of successful throws out of ten attempts without errors was recorded.

Second task: 'rolling ball into goal' or 'throwing bean bag into box' (for 5-6 or 7-10 year-old children, respectively). The child must roll a tennis ball on the floor between two stands to score a 'goal', or throw a bean bag into a target box on a floor form behind a marked line. The number of successful throws out of ten attempts without errors was recorded.

Static and dynamic balance

First task evaluating static balance: ‘one-leg balance’, ‘stork balance’ or ‘one-board balance’ (for 5-6, 7-8 or 9-10 year-old children, respectively). The child must stand on one foot, holding the arms at the sides, or placing the sole of the other foot against the side of the supporting knee and the hands on the hips, or balance on one foot on a balance board, respectively. The number of seconds up to 20 the child maintained balance was recorded.

Second task evaluating dynamic balance: ‘jumping over cord, ‘jumping in squares’ or ‘hopping in squares’ (for 5-6, 7-8 or 9-10 year-old children, respectively). The child must jump over the cord from a stationary position, or make five continuous jumps forward from a starting square to further five squares, or hops forward from square to square on one foot, respectively. The successfulness of the jump over the cord or the number of correct consecutive jumps/hops completed over five without performance errors was recorded.

Third task evaluating dynamic balance: ‘walking heels raised’, ‘heel-to-toe walking’ or ‘ball balance’. The child must walk along a line with heels raised without stepping off the line, or walk placing the heel of one foot against the toe of the other, or walk around the outside of two stands and return to the starting point while steadying a board with a ball in the middle, respectively. The number of steps performed by the child without leaving space between toe and heel or stepping off the line was recorded.

Test validity and reliability

The M-ABC has been proved valid and reliable for the entire age range of the test and for each age class. Test-retest reliability is very high (intraclass correlation coefficient [ICC] = .95 for all ages;.98, .95, .92 and .97 for 5-6, 7-8, 9-10 and 11-12 years, respectively) and convergent validity is strong (correlation of M-ABC with Bruininks-Oseretsky Test of Motor Proficiency, long version: Pearson r = .76 for all ages; .77, .76, .70 and .90 for 5-6, 7-8, 9-10 and 11-12 years, respectively; Croce et al., 2001).

Random Number Generation task

The Random Number Generation **(**RNG) task is a version validated for children aged five years and older (Towse and McIachlan, 1999) of the original RNG task developed for adults (Towse and Neil, 1998).

The TPI is a ratio between the real frequency of turning points between ascending and descending series of generated numbers (e.g., the change between the digits “2” and “5” in a hypothetical sequence “9, 7, 2, 5, 6, 8”) and their theoretical frequency in random responses. The Adj measures the relative frequency of pairs of adjacent ascending or descending numbers (e.g., 7-8 or 4-3) as compared to the total number of generated pairs. The Runs is an index of variability of the number of digits in successive ascending or descending runs. The Red reflects the frequency unbalance of generated digits when given numbers are used more frequently than expected based on the theoretical frequency of each digit in random responses. The Coupon is the number of digits generated until the entire set of digits alternatives (1 to 10) has been used. The MeanRG is the mean number of responses given until each digit reoccurs calculated for all digits throughout the whole sequence (e.g., in the sequence “2, 8, 4, 6, 2, 9, 7, 8”, the digits “2” and “8” reoccur with a mean gap equal to 4; for an extensive description of these indices see Audiffren et al., 2009).

The TPI, Adj, Runs, and the Red, Coupon, MeanRG are grouped in two three-factorial components (std. regression coefficients = .39 and .33, respectively; Miyake et al. 2000) with acceptable to high

internal consistency (ICC > .86 and > .82, respectively; Audiffren et al., 2009), reflecting the ability to inhibit mental counting routines and the ability to update information held in working memory, respectively.

Torrance Test of Creative Thinking

The TTCT Figural Form A is designed for individuals in kindergarten through graduate school and beyond. According to testing guidelines, the administrator invited the children to enjoy these activities and created a playful, not performance-oriented climate. The TTCT has a strong predictive validity, as its scores could explain 23% of the variance in creative production over a 40-year span (Cramond et al., 2005). Test-retest reliability coefficients (ICC), ranging .50 to .93, are deemed acceptable for group and research applications (Kim, 2006).

To ensure consistency in the evaluation of creativity in the cognitive and motor domain, we did not include indices of creative thinking different from fluency, flexibility, originality (i.e., we excluded ‘elaboration’ and two further indices added to the third TTCT edition, ‘abstractness of titles’ and ‘resistance to premature change’) but maintained flexibility, although in the third edition of the TTCT (Torrance and Ball, 1984), it was eliminated because of collinearity with fluency.

Children’s Outdoor Play assessment questionnaire

Children’s Outdoor Play assessment questionnaire (Veitch et al., 2009; Italian validation: Pesce et al., 2016b). Parents reported the number of days their child spent at least 10 minutes playing in locations such as their yard at home, a friend’s or neighbour’s yard, their street or court or footpath, a park or playground in out-of-school hours on weekdays (8 items on a five-point scale) and weekend days (8 items on a six-point scale) during a typical week.

Physical activity enjoyment questionnaire

The PA Enjoyment Questionnaire (Di Cagno *et al.*, 2006) showed a high internal consistency, as indexed by a good Cronbach’s alpha coefficient across the six items ($\alpha = .82$ in Di Cagno et al., 2006; $\alpha = .80$ in the present dataset).

References of Supplementary Material 1

Audiffren, M., Tomporowski, P.D., and Zagrodnik, J. (2009). Acute aerobic exercise and information processing: modulation of executive control in a Random Number Generation task. *Acta Psychol. (Amst).* 132, 85–95. doi: 10.1016/j.actpsy.2009.06.008

Bertsch, J. (1983). *Le créativitè motrice. Son evaluation et son optimisation dans la pédagogie des situations motrices a l’ecole – Manuel de tests [Motor creativity. Evaluation and optimization in the pedagogy of physical education – Test manual]*. Paris: INSEP.

Cramond, B., Matthews-Morgan, J., Bandalos, D., and Zuo, L. (2005). A report on the 40-year follow-up of the Torrance tests of creative thinking: alive and well in the new millennium. *Gift. Child Q.* 49, 283–291. doi:10.1177/001698620504900402

Croce, R.V., Horvat, M., and McCarthy, E. (2001). Reliability and concurrent validity of the movement assessment battery for children. *Percept. Mot. Skills*. 93, 275–280.

Henderson, S.E., and Sudgen, D.A. (1992). *Movement assessment battery for children*. London, UK: The Psychological Corporation. [Italian version: (2000). *Movement ABC—Batteria per la valutazione motoria del bambino,* eds. Eu. Mercuri and El. Mercuri. Firenze: Giunti O.S.].

Kim, K.H. (2006). Can we trust creativity tests? A review of the Torrance tests of creative thinking (TTCT). *Creat. Res. J.* 18, 3–14.

Miyake, A., Friedman, N.P., Emerson, M.J., Witzki, A.H., Howerter A, and Wager, T.D. (2000). The unity and diversity of executive functions and their contributions to complex "frontal lobe" tasks: a latent variable analysis. *Cogn. Psychol.* 41, 49–100. doi: 10.1006/cogp.1999.0734

Pesce, C., Masci, C., Marchetti, R., Vazou, S., Sääkslahti, A., and Tomporowski, P.D. (2016b). Deliberate play and preparation jointly benefit motor and cognitive development: mediated and moderated effects. *Front. Psychol.* 7:349. doi: 10.3389/fpsyg.2016.00349

Scibinetti, P., Tocci, N., and Pesce, C. (2011). Motor creativity and creative thinking in children: The diverging role of inhibition. *Creat. Res. J.* 23, 262–272. doi:10.1080/10400419.2011.595993

Schulz, J., Henderson, S.E., Sugden, D.A., and Barnett, A.L. (2011). Structural validity of the Movement ABC-2 test: factor structure comparisons across three age groups. *Res. Dev. Disabil.* 32, 1361–9. doi: 10.1016/j.ridd.2011.01.032

Torrance, E. P., and Ball, O. E. (1984). *The Torrance Tests of Creative Thinking Streamlined (revised) manual, Figural A and B*. Bensenville, IL: Scholastic Testing Service, Inc.

Towse, J.N., and McIachlan, A. (1999). An exploration of random generation among children. *Br. J. Dev. Psychol.* 17, 363–380. doi: 10.1348/026151099165348

Towse, J.N., and Neil, D. (1998). Analyzing human random generation behavior: a review of methods used and a computer program for describing performance. *Behav. Res. Meth. Instrum. Comput*. 30, 583–591, doi:10.3758/BF03209475

Veitch, J., Salmon, J., and Ball, K. (2009). The validity and reliability of an instrument to assess children's outdoor play in various locations. *J. Sc. Med. Sport.*12, 579–582. doi: 10.1016/j.jsams.2008.09.001

Wyrick, W. (1968). The development of a test of motor creativity. *Res. Q. Exerc. Sport.* 39, 756–765. doi: 10.1080/10671188.1968.10616608
